# Metagenomic and Metabolomic Insights Into the Mechanism Underlying the Disparity in Milk Yield of Holstein Cows

**DOI:** 10.3389/fmicb.2022.844968

**Published:** 2022-05-20

**Authors:** Abdulmumini B. Amin, Lei Zhang, JiYou Zhang, Shengyong Mao

**Affiliations:** ^1^Centre for Ruminant Nutrition and Feed Engineering Research, College of Animal Science and Technology, Nanjing Agricultural University, Nanjing, China; ^2^Laboratory for Gastrointestinal Microbiology, Jiangsu Key Laboratory of Gastrointestinal Nutrition and Animal Health, National Centre for International Research on Animal Gut Nutrition, College of Animal Science and Technology, Nanjing Agricultural University, Nanjing, China; ^3^Department of Animal Science, Federal University Dutse, Dutse, Nigeria

**Keywords:** Holstein cow, rumen metagenomics, metabolomics, milk yield, citric acid cycle

## Abstract

This study was conducted to investigate the metabolic mechanism underlying the disparity in the milk yield of Holstein cows. Eighteen lactating Holstein cows in their second parity and 56 (±14.81 SD) days in milking (DIM) were selected from 94 cows. Based on the milk yield of the cows, they were divided into two groups of nine cows each, the high milk yield group (HP) (44.57 ± 2.11 kg/day) and the low milk yield group (LP) (26.71 ± 0.70 kg/day). The experimental cows were fed the same diet and kept under the same management system for more than 60 days. Rumen metagenomics revealed that two *Archaea* genera, one *Bacteria* genus, eight *Eukaryota* genera, and two *Virus* genera differ between the HP and LP groups. The analysis of metabolites in the rumen fluid, milk, and serum showed that several metabolites differed between the HP and LP groups. Correlation analysis between the predominant microbiota and milk yield-associated metabolites (MP-metabolites) revealed that four *Bacteria* and two *Eukaryota* genera have a positive relationship with MP-metabolites. Pathway enrichment analysis of the differential metabolites revealed that five pathways were enriched in all the samples (two pathways in the milk, two pathways in the serum, and one pathway in the rumen fluid). Further investigation revealed that the low milk yield observed in the LP group might be due to an upregulation in dopamine levels in the rumen fluid and milk, which could inhibit the release of prolactin or suppress the action of oxytocin in the udder resulting in reduced milk yield. On the other hand, the high milk yield in the HP group is attributed to an upregulation in citrulline, and *N*-acetylornithine, which could be used as substrates for energy metabolism in the citric acid cycle and ultimately gluconeogenesis.

## Introduction

As the population of the world continues to rise, there is a corresponding increase in the demand for milk and milk products for the nourishment of the young and old in many countries (Boland and Hill, 2020). According to the F.A.O. (2021), the current global milk output is estimated at 921 million tons (an increase of 1.6% from that of the year 2020). Recent evidence suggests that the milk yield and milk protein yield (kg/cow/day) of dairy cows vary widely even when they are fed the same diet under the same management system (Wu et al., 2018, 2021; Xue et al., 2019, 2020). Several factors have been reported to influence milk production and quality in cows. These factors include; days in milking (DIM), breed, milking interval, type of feed, climate (Roy et al., 2020), and rumen microbiota (Tong et al., 2018).

The rumen is the largest stomach in matured ruminants and it contains a large diversity of beneficial microbiota that aid in the degradation of complex carbohydrates. These rumen microbes (*Archaea*, *Bacteria*, *Protozoa*, *Fungi*, and *Viruses*) associate with each other and with their environment. The fermentation activities of these microbes contribute immensely to the health, body maintenance, and production of ruminants through the synthesis of volatile fatty acids (VFAs), vitamins, and microbial protein (MCP) (Tong et al., 2018; Buitenhuis et al., 2019; Cunha et al., 2019; Wang et al., 2021; Wu et al., 2021). Furthermore, the disparity in the diversity and abundance of these rumen microbes is known to affect milk yield and milk protein yield in dairy cows (Tong et al., 2018; Xue et al., 2019). Studies on dairy cows under the same diet confirmed that the ruminal microbial communities (Tong et al., 2018; Mu et al., 2019) and fermentation parameters (Sofyan et al., 2017) of high-producing cows differs from those of low-producing cows. However, these investigations (Tong et al., 2018; Mu et al., 2019) were carried out *via* 16S rRNA sequencing, hence, the findings are limited to the bacterial composition and diversity. The application of mass spectrometry metabolomics and high-throughput metagenomic sequencing has helped researchers to uncover the metabolic pathways and detect biological markers underlying functions of microbial genomes and their host (Wang et al., 2018; Jing and Yan, 2020). Recent studies using metagenomics and metabolomics reveal an association between rumen microbiota and host metabolism in dairy cows with a high and low-milk protein yield (Xue et al., 2020). Hence, there is a need for further studies to uncover the metabolic mechanism underlying the variation in milk yield of dairy cows.

In this study, we speculated that the mechanism underlying the variation in milk yield among dairy cows fed the same diet might be affected by the composition of rumen microbiota and their association with the host metabolism. Thus, we explored rumen fermentation, rumen metagenomics (shotgun metagenomics), and metabolomics of serum, rumen fluid, and milk in high and low-producing dairy cows to fully understand the metabolic mechanism driving the variation in milk yield.

## Materials and Methods

### Ethics Statement

This study was conducted in strict adherence to the Animal Protection Law as stipulated in the Guide for the Care and Use of Laboratory Animals and approved by the Ethics Committee of Nanjing Agricultural University, Nanjing, China.

### Experimental Animals and Experimental Design

A total of 18 lactating Holstein cows on second parity and 56 (±14.81 SD) DIM were selected from 94 cows on a commercial dairy farm (Shanghai Yi Nan Dairy farm, Shanghai, China). All the cows were sired using artificial insemination and the semen used was purchased from the same company. The cows have been on the farm and fed the same diet for more than 60 days. The nutritional composition of the diets was based on the N.R.C. (2001) requirements for lactating Holstein cows (Table 1) and the average dry matter intake (DMI) for all the cows within the same row was recorded. All the animals were healthy and had access to clean drinking water *ad libitum*.

**TABLE 1:** Performance and milk composition of cows in HP and LP group.

Feed ingredients	Percentage
Corn flour	26.87
Soybean meal	11.52
Extruded soybean meal	3.43
Soybean hull	3.43
Cotton seed	6.86
Alfalfa hay	6.17
Oat hay	5.14
Alfalfa silage	3.50
Corn silage	25.70
Molasses	3.12
Yeast culture	0.12
Palm oil fat powder	0.12
Lysine	1.17
Methionine	0.04
Coated urea	0.31
*Premix* ^1^	2.50
**Total**	100.00
Nutrient composition	
Net energy of lactation (MJ/kg)	1.70
Crude protein	17.5%
Neutral detergent fiber	30.9%
Acid detergent fiber	18.9%
Ash	9.3%
Calcium	1.24%
Phosphorus	0.71%

*Premix^1^ contains 180 KIU/kg Vit A, 45 KIU/kg Vit D3, 1400 KIU/kg Vit E, 150 mg/kg copper methionine, 700 mg/kg zinc methionine, 170 mg/kg iron, 360 mg/kg copper, 680 mg/kg zinc, 910 mg/kg manganese, 10 g/kg methionine hydroxy analog, 4 mg/kg cobalt, 20 mg/kg iodine, 6 mg/kg selenium, 7 mg/kg selenium yeast, 1% total phosphorus, 5.1% calcium, 11.18% sodium chloride, and 4% magnesium.*

Milk samples were collected from all the cows three times daily (2 a.m., 10 a.m., and 5 p.m.) into a 50 ml tube containing a preservative (K_2_Cr_2_O_2_) for three consecutive days. The milk sample for each collection day was mixed at a ratio of 4:3:3 for the samples collected at 2 a.m., 10 a.m., and 5 p.m., respectively. The milk samples were then stored at 4°C while waiting to be sent for milk composition analysis. The milk samples were sent to the laboratory (Shanghai DHI company, Shanghai, China) within 2 days of collection for the determination of milk composition using near-infrared spectrometry (MilkoScan Minor FT120, Denmark). The determination of milk composition and somatic cell count was carried out separately for each collection day. Milk yield was recorded with the aid of a milk sampler (Waikato Milking Systems NZ Ltd., New Zealand). Another portion of the milk sample were collected from each cow into a 2 ml tube for the determination of milk metabolites. The milk samples were collected for three consecutive days and mixed to obtain a composite sample (day 1, day 2, and day 3) before analysis of milk metabolites. The milk samples were then stored at −20°C until required for analysis of metabolites. Based on the milk yield of the cows, 18 cows were selected from the 94 cows and divided into 2 groups of 9 cows each. To ensure that cows in the two groups have significantly different milk yields, nine cows with the highest milk yield (44.57 ± 2.11 kg/day) were placed in the high-producing (HP) group while nine cows with the lowest milk yield (26.71 ± 0.70 kg/day) were allotted to the low-producing (LP) group. The selected cows have an average weight of 639.17 ± 17.49 kg.

### Collection of Rumen Content and Analysis

Rumen content was collected from each of the 18 cows before morning feeding into 50 ml tubes (×2) using an oral rumen tube. To maintain the purity of rumen content and avoid mixing with saliva, the first 150 ml of rumen content collected was discarded. The rumen content (10 ml) to be used for metagenomics and MCP determination was immediately stored in liquid nitrogen. Another portion of the rumen content was filtered through four layers of sterile cheesecloth into 5 ml tubes (three tubes) to be used for the detection of VFAs, ammonia nitrogen (NH_3_-N), and rumen fluid metabolites. For the rumen fluid sample meant for VFA analysis, 1 ml of 250 g/L metaphosphoric acid was added to the 5 ml rumen fluid filtrate. For rumen fluid filtrate meant for the determination of NH_3_-N, exactly 1 ml of 20 g/L (w/v) H_2_SO_4_ was added to the 5 ml and NH_3_-N was detected using the phenol-hypochlorite reaction (Weatherburn, 1967). The samples were stored at −20° until needed for the determination of rumen fluid metabolites and rumen fermentation parameters. The analysis of VFAs of the ruminal fluid samples was performed with gas chromatography (GC-2014B, Shimadzu, Japan) equipped with a capillary column (film thickness: 30 m × 0.32 mm × 0.25 μm, column temperature: 110°C). The temperatures of both the injector and detector were preset at 180°C.

Microbial protein was determined using the Bradford protein assay. Prior to the protein determination, the rumen fluid bacterial cells were extracted as described by Makkar et al. (1982). Briefly, about 10 ml of rumen fluid was stirred with a magnetic stirrer at 400 rpm for 45 s to remove the microbes attached to the feed particles and then centrifuged for 5 min at 408 × *g* to remove the protozoa. Five milliliters aliquot was taken from each of the samples and centrifuged again for 20 min at 25,000 × *g*. The supernatants were discarded, and pellets were washed with distilled water and centrifuged again at 25,000 × *g* for 20 min. The supernatants were discarded again, and the cells were suspended in 15 ml of 0.25 N NaOH and boiled for 10 min in a water bath before centrifuging at 25,000 × *g* for 30 min. Finally, the supernatant was collected and MCP was determined from the supernatant using the Bradford protein assay (Bradford, 1976).

Exactly 5 μl of the supernatant for each sample and 5 μl of a standard (Bovine serum albumin) were taken and transferred into microplate wells. Exactly 250 μl of 1× dye reagent (dye solution + methanol and phosphoric acid) was added to each of the wells and mixed thoroughly using a pipette by pressing the plunger repeatedly. A new pipette tip was used for each sample. The mixture was allowed to incubate at room temperature for 1 h and the absorbance was measured with a microplate reader (BioTek, Synergy H1, United States) set at 595 nm. The MCP concentration of the rumen fluid is estimated using a standard curve by plotting the 595 nm values on the *y*-axis versus their concentration in μg/ml on the *x*-axis.

### Collection of Blood Samples and Detection of Serum Biochemical Indices

Blood samples to be used for detection of serum metabolites were collected once from each cow *via* the tail vein 1 h before morning feeding into a 5 ml tube containing heparin. Another set of 5 ml tubes without anticoagulant was used to collect blood samples for detection of serum biochemical indices. A warm water bath (37°C) was used to keep the clotted blood for at least 20 min and then all the blood samples collected were centrifuged at 3000 × *g* for 15 min. The serum was collected with a pipette into 2 ml tubes and stored in a refrigerator at −80° until required for detection of serum biochemical parameters and serum metabolites. Serum biochemical indices were detected by injecting the serum samples into the tubes of an automated clinical chemistry analyzer (model CA-800) at the Jiangsu Hospital of Traditional Chinese Medicine, Nanjing, China.

### Analysis of Metabolites in the Rumen Fluid, Serum, and Milk

The analysis of metabolites in the rumen fluid, serum, and milk was carried out at Shanghai cluster Biotechnology Co., Ltd., Shanghai, China. The stored samples (rumen fluid, serum, and milk) were slowly thawed on ice, and 100 μl of each sample was transferred into a 1.5 ml centrifuge tube. Exactly 300 μl of methanol was added and the solution was vortexed for 30 s. The solution was incubated for 1 h at 40°C and vortexed again for 30 s before centrifuging (12,000 rpm) for 15 min at 4°C. Exactly 200 μl of the supernatant was taken and mixed with 5 μl of internal standard (0.14 mg/ml dichlorophenylalanine) before transferring the solution into an injection vial.

The detection of metabolites was performed using an LC–MS (Thermo, ultimate 3000lc). The machine (LC–MS) is equipped with a C18 column [hyper gold C18 (100 × 2.1 mm, 1.9 μm)] preset at a flow rate of 0.3 ml/min and a temperature of 40°C. The mobile phase is made up of A (water, 5% acetonitrile, and 0.1% formic acid) and B (acetonitrile and 0.1% formic acid). The elution process of the mobile phase is shown in Supplementary Table 1. Exactly 4 μl of each sample was injected into the autosampler, which was preset at 4°C. The spray voltage was set at 3.0 kV for the positive and 3.2 kV for the negative modes while other parameters were the same for both the positive and negative modes (Sheath gas flow rate 15 arb heater temperature 300°C, and capillary temperature 350°C). The extraction of the raw peak, filtration and calibration of the data baseline, identification of peak, alignment of the peak, integration of the peak area, and deconvolution analysis were performed on the LECO-Fiehn Rtx5 database (Kind et al., 2009). Metabolite profiling and identification were performed as described by Wang et al. (2018).

### Collection of Feed Samples and Analysis

Feed samples were randomly collected into a plastic bag at different points during feeding and stored at −80°C until required for analysis of chemical composition. The crude protein, crude fiber, crude fat, and ash were analyzed according to the official analytical methods of the A.O.A.C. (1991) whereas the acid detergent fiber (ADF) and neutral detergent fiber (NDF) were determined following the procedure of Van Soest et al. (1991). The feed ingredients and their nutrient composition are provided in Table 1.

### DNA Extraction and Sequencing

Microbial DNA extraction was carried out using the E.Z.N.A.^®^ stool DNA Kit while strictly adhering to the procedures stipulated by the company (Omega Bio-Tek, Norcross, GA, United States). In brief, the rumen samples were previously obtained from each cow in duplicates *via* oral rumen tube and were mixed to get a composite sample before storage in liquid nitrogen. Frozen rumen samples were thawed at 4°C, centrifuged at low speed to obtain the particulate matter and about 200 mg of the composite sample from each cow was collected into a disruptor tube. Exactly 725 μl of a lysis buffer was added and the samples were vortexed on a bead-ruptor for 3--5 min to lyse the samples. DNA was extracted from each sample following the guidelines of the DNA Kit’s manufacturer (Omega Bio-Tek, Norcross, GA, United States) and DNA concentration was measured with a NanoDrop ND-1000 Spectrophotometer (Thermo Scientific, United States). The construction of the metagenomic shotgun sequencing libraries and the DNA sequencing were done at Shanghai Biozeron Biological Technology Co., Ltd. (Shanghai, China). A TruSeq DNA PCR-Free library preparation kit (Illumina, San Diego, CA, United States) was used to construct sequencing libraries (length of about 350 bp) from the extracted DNA. An Illumina NovaSeq instrument was used for sequencing all the samples. Thereafter, raw sequence reads were subjected to quality trimming using Trimmomatic^1^ following the procedures described by Bolger et al. (2014). Good quality reads that passed the quality trimming were mapped against *Bos taurus* ARS-UCD1.2, using the BWA mem algorithm (parameters: -M -k 32 -t 16^2^). Good quality reads devoid of host genome contaminations (clean reads) were used for phylogenetic annotation.

### Phylogenetic Annotation and Gene Prediction

Clean reads obtained from each sample were further subjected to taxonomic classification with Kraken2 software (Wood and Salzberg, 2014), which uses the National Centre for Biotechnology Institute (NCBI) RefSeq database represented by a series of clade-specific *k*-mers in which the clean reads are matched against. The classification was done at seven phylogenetic levels which are domain, phylum, class, order, family, genus, species, or unclassified. Bracken^3^ was used to calculate the abundance of each taxonomy. The functional annotations of the microbiota were obtained from the Kyoto Encyclopedia of Genes and Genomes (KEGG) database^4^ using the KEGG Orthology Based Annotation System (KOBAS version 2.0).^5^

A set of contigs was obtained from the clean reads using MegaHit (parameters: –min-contig-len 500) (Li et al., 2015). The prediction of the open reading frames (ORFs) of assembled contigs was performed with Prodigal software (v2.6.3) (Hyatt et al., 2010), while CD-HIT (parameters: -n 9 -c 0.95 -G 0 -M 0 -d 0 -aS 0.9 -r 1) (Fu et al., 2012) was used for clustering to obtain a unique-gene set. Within a unique gene set, the longest sequence in each of the clusters is the representative sequence of each gene. Salmon software (Patro et al., 2017) was used to obtain the number of reads for each gene, and the gene abundance of all the samples was calculated using the formula below (Li et al., 2014):


(1) A⁢b⁢(S)⁢=⁢A⁢b⁢⁢(U)⁢+⁢A⁢b⁢⁢(M)

(2) $$A\,b\,\left(U\right)=\sum_{i=1}^{M}1/l$$

(3) $$A\,b\,\left(M\right)=\sum_{i=1}^{M}\left(\mathrm{Co}*1\right)/l$$

(4) $$C\,o=\frac{A\,b\,\left(U\right)}{\sum_{i=1}^{N}A\,b\,\left(U_{i}\right)}$$

where, *Ab*(*S*), gene abundance; *Ab*(*U*), single-mapping reads abundance; *Ab*(*M*), multi-mapping reads abundance; *l*, length of gene sequence.

To obtain the list and functional annotations of Carbohydrate-Active Enzymes (CAZymes), the gene sequence was converted into an amino acid sequence, then DIAMOND software (Buchfink et al., 2015) was used to align the sequence to the CAZymes database (Lombard et al., 2014). The sequences of rumen microbial metagenome were submitted to the Sequence Read Archive (SRA) of the NCBI with the accession number PRJNA748874.^6^

### Statistical Analysis and Network Analysis

At the commencement of the study, a power analysis was conducted with SAS (9.4) to determine the number of cows per group, and the results revealed that a minimum of seven cows per group is required to obtain a statistical power of 0.84.

The results obtained from rumen fermentation parameters, DMI, milk composition, and yield were analyzed with the Student’s *T*-test using the IBM SPSS (v20). The results were considered significant at *P* < 0.05. The results of the metabolites from milk, rumen fluid, and serum were first subjected to principal component analysis (PCA) and partial least square discriminant analysis (PLS-DA) using SIMCA (13.0). Those metabolites with variable important (VIP) scores greater than 1 were further analyzed using the Student’s *T*-test (*P* < 0.05) in IBM SPSS (v20). Differential metabolites between the HP and LP group were matched to the KEGG pathway (*B. taurus* library) on MetaboAnalyst 5.0.^7^ The rumen microbiota for all the domains (*Archaea*, *Bacteria*, *Eukaryota*, and *Virus*) at phylum and genus levels, the abundance of genes encoding CAZymes, KEGG genes, and functions were filtered by relative abundance greater than 0.1% in at least 1 sample before subjecting them to the linear discriminant analysis effect size (LEfSe) (Segata et al., 2011) using web-based software.^8^ LEfSe uses the Kruskal–Wallis test to identify significantly different parameters before subjecting them to linear discriminant analysis (LDA) to estimate the effect size of each differential parameter. The taxa, CAZymes, KEGG genes, and functions were declared significant at *P* < 0.05 and LDA > 2.

The PCA, principal coordinate analysis (PCoA), and the permutational multivariate analysis of variance (PERMANOVA) of the rumen microbiota were conducted using the Vegan package in R software (4.05). Spearman’s correlation analysis was conducted using IBM SPSS (v20) to identify the relationship among the metabolites (milk, rumen fluid, and serum), predominant microbiota (relative abundance >1%), fermentation parameters, and milk yield. The correlation between metabolites (milk, rumen fluid, and serum) and milk yield was conducted and those metabolites with a strong positive association with milk yield (*R* > 0.6, *P* < 0.05) were tagged MP-metabolites (Supplementary Table 10). Furthermore, the predominant rumen microbiota were correlated (*R* > 0.6, *P* < 0.05) with the MP-metabolites (milk, rumen fluid, serum) (Supplementary Table 12), and the fermentation parameters in the rumen (*R* > 0.6, *P* < 0.05) (Supplementary Table 13). All the significant correlation networks were visualized using Cytoscape (3.8.2).

## Results

### Milk Composition and Rumen Fermentation Parameters

**FIGURE 1:**
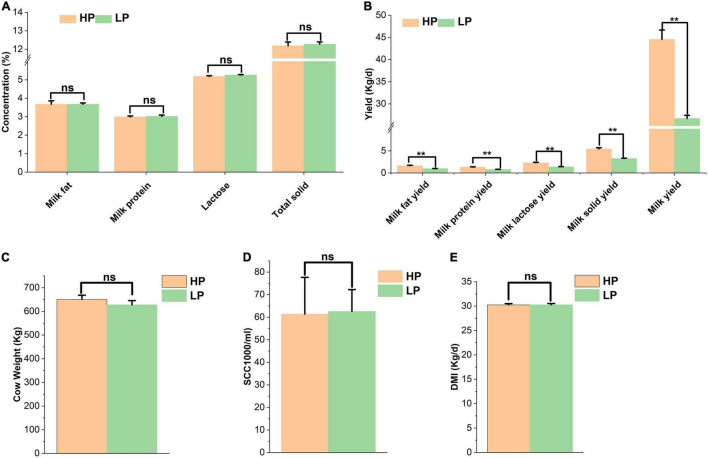
**(A)** Percentage of milk components of cows in the HP and LP groups. **(B)** The yield of milk components of cows in the HP and LP groups. **(C)** Average weight of cows in the HP and LP groups. **(D)** Somatic cell count in the HP and LP groups. **(E)** The average DMI of cows in the HP and LP groups. ns: indicates not significant (*p* > 0.05), **indicates significant difference (*p* < 0.01).

The results obtained from milk composition analysis and somatic cell count showed that the milk yield (kg/day), milk fat yield (kg/day), milk protein yield (kg/day), lactose yield (kg/day), and milk solid yield (kg/day) were significantly higher (*P* < 0.01) in the HP group as compared to the LP group. The average milk yield of the HP group is 44.57 kg/cow per day whereas that of the LP group is 26.71 kg/cow per day. The milk protein yield of the HP group is 1.33 kg/cow per day while that of the LP group is 0.81 kg/cow per day (Table 2 and Figure 1).

**TABLE 2:** Milk composition of cows in HP and LP group.

Item	HP	LP	SEM	*P*-value
DMI (kg/day)	30.26	30.28	0.26	0.97
Weight (kg)	650.67	627.67	17.49	0.53
Milk yield (kg/cow/day)	44.57	26.71	2.42	<0.01
DIM	54.56	49.38	3.43	0.48
Milk fat (%)	3.68	3.68	0.09	1.00
Milk protein (%)	2.99	3.02	0.04	0.69
Lactose (%)	5.19	5.26	0.02	0.10
Total solid (%)	12.20	12.28	0.11	0.72
SCC (1000/ml)	61.41	62.63	9.17	0.95
Milk fat yield (kg/cow/day)	1.64	0.98	0.10	<0.01
Milk protein yield (kg/cow/day)	1.33	0.81	0.07	<0.01
Lactose yield (kg/cow/day)	2.31	1.40	0.12	<0.01
Milk solid yield (kg/cow/day)	5.43	3.28	0.29	<0.01

*DMI, dry matter intake; DIM, days in milking; SCC, somatic cell count.*

The results of rumen fluid analysis revealed that the percentages of the rumen VFAs (acetate, propionate, butyrate, isobutyrate) and the acetate: propionate ratio did not differ significantly (*P* > 0.05) between the HP and LP groups. However, the percentages of valerate and isovalerate were found to be significantly higher (*P* < 0.05) in the HP group whereas, the LP group recorded a significantly higher (*P* = 0.029) proportion of ruminal NH_3_-N (mg/dl). The average concentration of NH_3_-N observed in the HP group is 12.53 mg/dl while that of the LP group is 19.60 mg/dl (Table 3).

**TABLE 3:** Rumen fermentation parameters of HP and LP group.

Item	HP	LP	SEM	*P*-value
Acetate (%)	61.03	62.47	0.48	0.14
Propionate (%)	22.46	22.03	0.57	0.72
Butyrate (%)	12.69	12.08	0.33	0.37
Acetate: propionate	2.72	2.91	0.09	0.32
Isobutyrate (%)	0.82	0.79	0.02	0.31
Isovalerate (%)	1.48	1.38	0.03	0.047
Valerate (%)	1.52	1.27	0.06	0.019
Total VFA (mmol/L)	109.16	116.95	3.66	0.30
NH_3_-N (mg/dl)	12.53	19.60	1.67	0.03
MCP (mg/dl)	67.41	59.53	3.01	0.62

### Sequence Classification

The metagenomic sequencing of the 18 samples generated a total of 1,299,004,492 raw reads with an average of 72,166,916.22 reads ± 2,167,434.65 per sample. After filtering and removing host genes, the total clean reads recorded were 1,178,340,750 with an average of 65,463,375 ± 1,922,941.62 reads per sample (Supplementary Table 2).

### Beta Diversity of Rumen Microbial Communities in the HP

The PCoA was carried out based on Bray Curtis distance metrics and the plot represents 70% of the variation (Axis 1 and Axis 2) yet no clear separation between the HP and the LP group. Similarly, the PCA showed 82% of the variation (PC 1 and PC 2) yet no clear distinction between the HP and the LP group. However, the samples in the LP as observed in both the PCoA (Supplementary Figure 3) and PCA (Supplementary Figure 4) plots tend to cluster closer to each other than those of the HP group. Furthermore, PERMANOVA was performed on the abundance of *Archaea*, *Bacteria*, *Eukaryota*, and *Viruses*. The results revealed that none of the domains differed significantly (*P* > 0.05) between the HP and the LP groups (Supplementary Table 3 and Supplementary Figure 1).

### Taxonomic Classification of Rumen Metagenome in the HP and LP Group

**FIGURE 2:**
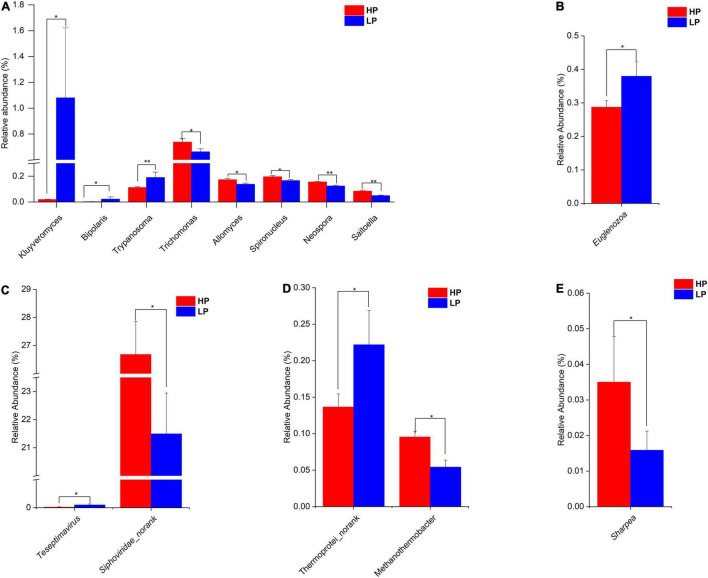
**(A)** Relative abundance of differential *Eukaryota* genera (*P* < 0.05, LDA > 2) in the HP and LP groups. **(B)** Relative abundance of differential *Eukaryota* phylum (*P* < 0.05, LDA > 2) in the HP and LP groups. **(C)** Relative abundance of differential *Virus* genera (*P* < 0.05, LDA > 2) in the HP and LP groups. **(D)** Relative abundance of differential *Archaea* genera (*P* < 0.05, LDA > 2) in the HP and LP groups. **(E)** Relative abundance of differential *Bacteria* genus (*P* < 0.05, LDA > 2) in the HP and LP groups. *indicates significant difference (*p* < 0.05), **indicates significant difference (*p* < 0.01).

After the taxonomic classification, a total of 3982 genera and 212 phyla were obtained in all 4 domains. The *Archaea* had 22 and 147 phyla and genera, respectively. In the *Bacteria*, 146 phyla and 3033 genera were found whereas the *Eukaryota* had 43 and 595 phyla and genera, respectively. However, the *Viruses* were unclassified at the phylum level but there were 207 genera identified. In the *Archaea*, the Phylum *Euryarchaeota* predominated with a relative abundance of 96.74 and 96.68% in the HP and LP groups, respectively (Supplementary Table 5A and Supplementary Figure 5B). At the genus level, *Methanobrevibacter* was the most abundant genera with a relative abundance of 83.70% in the HP group and 82.02% in the LP group (Supplementary Table 5B and Supplementary Figure 5A). At the genus level, *Methanothermobacter* was significantly higher (*P* = 0.012, LDA > 2) in the HP group while *Thermoprotei_norank* was found to be significantly higher (*P* = 0.047, LDA > 2) in the LP group (Figure 2D).

Among the *Bacteria*, *Bacteroidetes* are the most abundant phylum in both groups with HP and LP occupying 68.34 and 71.82%, respectively Supplementary Figure 5D. *Firmicutes* also represent a large portion of the rumen *Bacterial* phyla with the HP group occupying 21.43% while the LP group representing 17.73% (Supplementary Table 6A). For the *Bacterial* genus, *Prevotella* topped the list of predominant genera with the HP and LP groups having a relative abundance of 50.63 and 53.67%, respectively Supplementary Figure 5C. None of the *Bacterial* Phyla was found to differ significantly between the HP and LP groups. However, at the genus level, the relative abundance of the *Sharpea* was significantly higher (*P* = 0.047, LDA > 2) in the HP group when compared to the LP group (Figure 2E).

In the *Eukaryota*, the *Ciliophora* is the most abundant Phylum which represents about 62–63% in both the HP and the LP group (Supplementary Table 7A and Supplementary Figures 6A,B). At the phylum level, the relative abundance of *Euglenozoa* was significantly higher (*P* = 0.015, LDA > 2) in the LP group (Figure 2B and Supplementary Figure 7). As for the genera, eight genera were found to differ significantly between the HP and LP groups (Figure 2A). The HP group recorded a significantly higher abundance of the genera *Trichomonas* (*P* = 0.047, LDA > 2), *Spironucleus* (*P* = 0.038, LDA > 2), *Allomyces* (*P* = 0.019, LDA > 2), *Saitoella* (0.003, LDA > 2), and *Neospora* (*P* = 0.005, LDA > 2). On the other hand, the genera *Kluyveromyces* (*P* = 0.047, LDA > 2), *Trypanosoma* (*P* = 0.007, LDA > 2), and *Bipolaris* (0.046, LDA > 2) were found to be significantly higher in the LP group as compared to the HP group.

**FIGURE 3:**
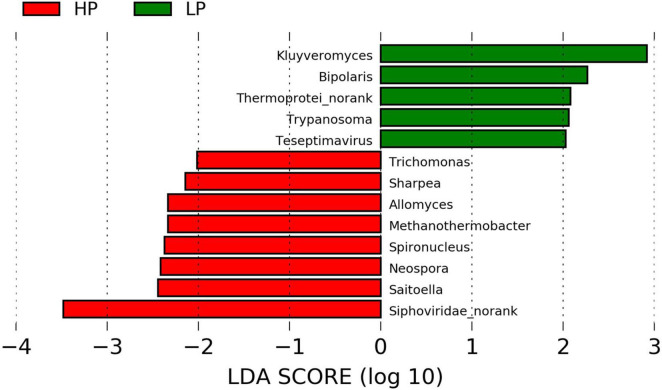
Linear discriminant analysis effect size plot of differential microbial genera (*P* < 0.05, LDA > 2) in the HP and LP groups.

In the Viruses, the most abundant genus is the *Podoviridae_norank* which represents 37.77 and 36.46% in the HP and LP group, respectively (Supplementary Table 8 and Supplementary Figure 6C). The next most abundant genus is the *Siphoviridae_norank* and this genus was found to be significantly higher (*P* = 0.031, LDA > 2) in the HP group (26.68%) when compared to the LP group (21.49%). However, the LP group recorded a significantly higher abundance (*P* = 0.024, LDA > 2) of *Teseptimavirus* (Figures 2C, 3).

### Functional Annotation of Rumen Microbiome in HP and LP Group

**FIGURE 4:**
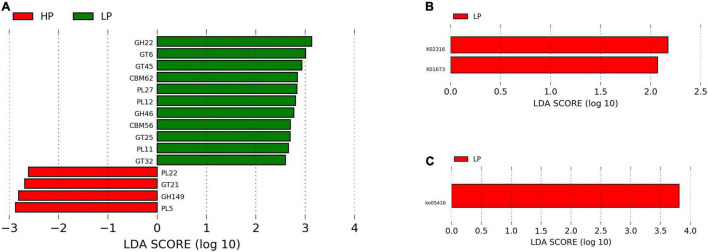
**(A)** Linear discriminant analysis effect size plot of differential CAZymes (*P* < 0.05, LDA > 2) in the HP and LP groups. **(B)** LefSe plot of differential KEGG genes (*P* < 0.05, LDA > 2) in the HP and LP groups. **(C)** LefSe plot of differential KEGG pathways (*P* < 0.05, LDA > 2) in the HP and LP groups.

The functional annotation of the rumen microbiome was defined by the KEGG profiles. A total of 368 level-3 KEGG pathways were identified. After subjecting the KEGG profiles to LEfSe, ko05416 (*Viral* myocarditis) was significantly upregulated (*P* = 0.012, LDA > 2) in the LP group (Figure 4C).

### Activities of Carbohydrate-Active Enzymes

A total of 342 genes associated with CAZymes belonging to the following families were identified. These are; 132 glycoside hydrolases (GHs), 30 polysaccharide lyases (PL), 80 carbohydrate-binding modules (CBMs), 16 carbohydrate esterases (CEs), 74 glycosyltransferases (GTs), and 10 auxiliary activities (AAs). However, only 15 CAZymes were found to differ significantly (*P* < 0.05, LDA > 2) between the HP and LP groups. The HP group recorded a significantly higher (*P* < 0.05, LDA > 2) expression of CAZymes belonging to GH149, GT21, PL22, and PL5. However, in the LP group, several CAZymes (CBM56, CBM62, GH22, GH46, GT25, GT32, GT45, GT6, PL11, PL12, and PL27) were significantly enriched (*P* < 0.05, LDA > 2) (Supplementary Table 4 and Figure 4A).

### Abundance of Genes Encoding Enzymes Involved in the Metabolic Pathways

A total of 2720 unique KEGG Orthology (KO) genes were found and subjected to LEfSe. Two genes (K02316 and K01673) were identified to differ significantly (*P* < 0.05, LDA > 2) between the HP and LP groups. After matching the identified genes to the KO database, K02316 (*dnaG*) was identified to belong to the class of transferases while K01673 (*cynT*) is found to be involved in nitrogen metabolism. Interestingly, both K02316 and K01673 were enriched in the LP group (Figure 4B).

### Differences in Rumen Fluid, Serum, and Milk Metabolites of Cows in HP and LP Groups

#### Serum Metabolites

**FIGURE 5:**
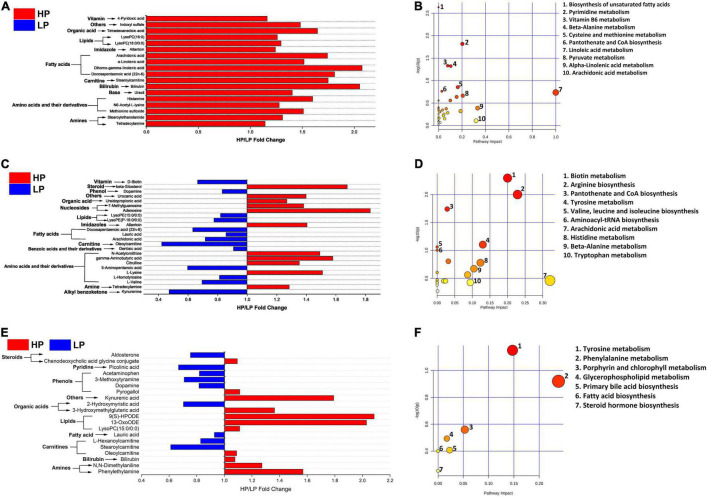
**(A)** Fold change (HP/LP) of significantly different (VIP > 1, FDR < 0.05) serum metabolites between the HP and LP cows. **(B)** The metabolic pathway impacted by serum metabolites in the HP and LP cows. **(C)** Fold change (HP/LP) of significantly different (VIP > 1, *P* < 0.05) milk metabolites between HP and LP cows. **(D)** The metabolic pathway impacted by milk metabolites in the HP and LP cows. **(E)** Fold change (HP/LP) of significantly different (VIP > 1, *P* < 0.05) rumen metabolites between the HP and LP cows. **(F)** The metabolic pathway impacted the rumen metabolites between the HP and LP cows. In the metabolic pathways, the bigger the circle, the higher the pathway impact, while the darker the color, the greater the changes in the metabolites in the corresponding pathway.

A total of 311 metabolites were identified in serum and then subjected to several filtering processes to ascertain the differential metabolites (VIP > 1, *P* < 0.05) between the HP and LP groups. Thereafter, a total of 78 serum metabolites were identified to differ significantly (VIP > 1, *P* < 0.05) between the HP and the LP group (Supplementary Table 9A and Figure 5A). The differential metabolites were mapped to the KEGG pathway (*B. taurus*) using web-based software (MetaboAnalyst 5.0). A total of 36 pathways were identified (Figure 5B) out of which only 2 pathways (pyrimidine metabolism and beta-alanine metabolism) were significantly enriched. Interestingly, both pyrimidine metabolism (*P* = 0.014, pathway impact = 0.20) and beta-alanine metabolism (*P* = 0.045, pathway impact = 0.10) were significantly enriched in the HP group as compared to the LP group (Supplementary Table 11A).

#### Milk Metabolites

Similar to the rumen fluid and the serum, 311 metabolites were identified in the milk samples and these were subjected to several filtering stages to identify the differential metabolites (VIP > 1, *P* < 0.05) between the HP and LP groups. After the screening stages, a total of 24 milk metabolites were observed to differ significantly (VIP > 1, *P* < 0.05) between the HP and the LP group (Supplementary Table 9C and Figure 5C). These were further mapped to the KEGG pathway (*B. taurus*) on a web platform (MetaboAnalyst 5.0). A total of 20 pathways were identified in the milk, out of which 2 (arginine biosynthesis and tyrosine metabolism) were significantly enriched (*P* < 0.05). Interestingly, the arginine biosynthesis pathway was significantly upregulated (*P* = 0.003, pathway impact = 0.23) in the HP group while the tyrosine metabolism (*P* = 0.02, pathway impact = 0.13) is enriched in the LP group (Figure 5D and Supplementary Table 11B).

#### Rumen Fluid Metabolites

In the rumen fluid, 311 metabolites were identified and these were subjected to several filtering stages to identify the differential metabolites (VIP > 1, *P* < 0.05) between the HP and LP groups. After the screening stage, 20 rumen metabolites were found to differ significantly (VIP > 1, *P* < 0.05) between the HP and the LP group (Supplementary Table 9B and Figure 5E). All the differential metabolites in the rumen fluid were matched to the KEGG pathway (*B. taurus*) using MetaboAnalyst 5.0. A total of seven pathways (Figure 5F) were identified in the rumen fluid but only one pathway (tyrosine metabolism) was significantly enriched (*P* = 0.02, pathway impact = 0.13) in the LP group as compared to the HP group (Supplementary Table 11C).

#### Association Between Differential Metabolites in the Rumen Fluid, Milk, and Serum With Milk Yield

To identify the metabolites with a positive association with milk yield, the differential metabolites (VIP > 1, *P* < 0.05) in the rumen fluid, milk, and serum were correlated (Spearman, *R* > 0.6, *P* < 0.05) with milk yield (Supplementary Table 10). A total of 45 metabolites (6 rumen metabolites, 5 milk metabolites, and 34 serum metabolites) were identified to have a high and significant positive correlation (Spearman *R* > 0.6, *P* < 0.05) with milk yield, hence called MP-metabolites.

#### Serum Biochemical Indices

The serum biochemical indices of cows in the HP and LP groups are presented in Table 4. The aspartate aminotransferase (*P* = 0.034), albumin (*P* = 0.003) and total cholesterol (*P* = 0.03) were found to be significantly higher in the HP when compared to the LP group. All other serum biochemical parameters were statistically similar between the two groups.

**TABLE 4:** Serum biochemical indices of cows in the HP and LP groups.

Item	HP	LP	SEM	*P*-value
AST (U/L)	69.78	59.00	2.61	0.034
ALT (U/L)	23.00	21.00	1.18	0.412
TP (g/L)	79.23	76.28	1.39	0.301
ALB (g/L)	39.00	35.60	0.63	0.003
GLOB (g/L)	40.24	40.68	1.53	0.892
ALB/GLOB	0.97	0.90	0.04	0.384
ALP (U/L)	59.33	59.22	2.63	0.984
CK (U/L)	176.33	134.56	18.01	0.258
LDH (U/L)	982.33	987.11	38.35	0.953
Urea (mmol/L)	5.02	4.73	0.18	0.435
Creatine (mmol/L)	70.34	73.67	1.92	0.403
Glucose (mmol/L)	2.64	2.61	0.13	0.897
Uric acid (mmol/L)	60.11	58.78	3.15	0.84
Total cholesterol (mmol/L)	5.32	4.05	0.30	0.03
Triglycerides (mmol/L)	0.15	0.14	0.00	0.909
HDL-C (mmol/L)	2.04	1.77	0.07	0.056
LDL-C (mmol/L)	1.52	1.15	0.10	0.057

*AST, aspartate aminotransferase; ALT, alanine aminotransferase; TP, total protein; ALB, albumin; ALP, alkaline phosphatase; GLOB, globulin; ALB/GLOB, albumin/globulin; ALP, alkaline phosphatase; CK, creatine kinase; LDH, lactic acid dehydrogenase; HDL-C, high density lipoprotein; LDL-C, low density lipoprotein.*

### Relationship Between Predominant Rumen Microbiota With Rumen Fermentation, Milk Yield, and MP-Metabolites in the Rumen Fluid, Milk, and Serum

**FIGURE 6:**
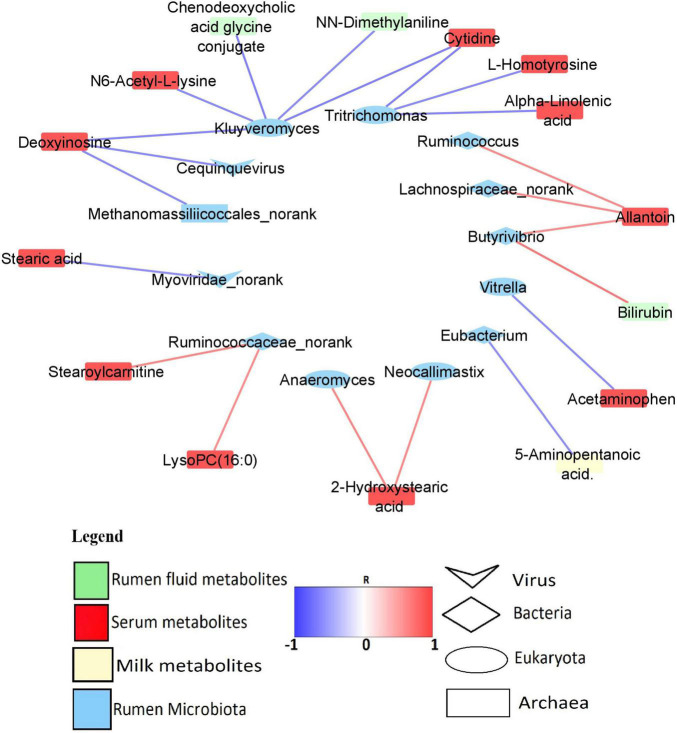
Network of significant correlation (*R* > 0.6/*R* < –0.6, *P* < 0.05) between the predominant rumen microbiota and MP-metabolites.

Predominant rumen microbiota were correlated with milk yield and all the 45 MP-metabolites in the rumen, serum, and milk (Supplementary Table 12). However, only 1 MP-metabolite in the milk, 3 MP-metabolites in the rumen, and 17 serum metabolites were found to strongly associate (*R* > 0.6/*R* < −0.6, *P* < 0.05) with the rumen microbiota. The rumen microbes with high and significant correlation (*R* > 0.6/*R* < −0.6, *P* < 0.05) with the MP-metabolites were considered as being associated with milk yield. A total of one *Archaeon* (*Methanomassiliicoccales_norank*), five *Bacteria* genera (*Eubacterium*, *Butyrivibrio*, *Lachnospiraceae_norank*, *Ruminococcus*, and *Ruminococcaceae_norank*), five *Eukaryota* genera (*Kluyveromyces*, *Tritrichomonas*, *Neocallimastix*, *Anaeromyces*, and *Vitrella*), and two *Viruses* (*Myoviridae_norank*, and *Cequinquevirus*) were identified to have a strong association (*R* > 0.6/*R* < −0.6, *P* < 0.05) with MP-metabolites (Figure 6). In addition, the correlation between predominant microbiota in the rumen and rumen fermentation parameters revealed a strong and positive association (*R* > 0.6, *P* < 0.05) between fermentation parameters and some genera from *Bacteria*, *Archaea*, *Eukaryota*, and *viruses* (Supplementary Table 13 and Supplementary Figure 2).

## Discussion

In the present study, we selected 18 lactating cows in their second parity from 94 cows and divided them into 2 groups (based on their milk yields), HP (44.57 ± 2.11 kg/day) and LP (26.71 ± 0.70 kg/day). The HP group had a significantly higher milk yield, milk protein yield, lactose yield, milk fat yield, and milk solid yield than the LP group. This is one of the few studies that employed metagenomics and metabolomics to study the relationship between rumen microbiota and host metabolism in high and low milk-yielding cows. Since the genetic production traits interact with the environmental factors (physical, chemical, and biological) to affect phenotypic traits (Kiplagat et al., 2012), the role of rumen microbiota and host metabolites in driving the milk yield is paramount.

The analysis of rumen fluid fermentation revealed no significant difference in VFAs except the percentages of valerate and isovalerate which were found to be significantly higher in the HP group. An increased ruminal concentrations of valerate and isovalerate were reported to stimulate the synthesis of MCP *in vitro* (Piva et al., 1988) and decrease the concentration of ruminal NH_3_-N in steers (Liu et al., 2009). The LP group recorded a significantly higher proportion of ruminal NH_3_-N (mg/dl) (Table 3). Interestingly, rumen content metagenomics revealed that the gene K01673 (*cynT*) was also found to be enriched in the LP group (Figure 3B). This gene (K01673) encodes the activities of carbonic anhydrase (EC: 4.2.1.1), an enzyme known to be associated with urea synthesis (Dodgson and Forster, 1986; Häussinger et al., 1986). Usually, a higher NH_3_-N concentration in the rumen is expected to increase the synthesis of MCP which serves as an additional protein source for the dairy cow, thus increasing milk yield (Calsamiglia et al., 2010). However, in our study, the higher NH_3_-N observed in the LP group could be because of less efficient utilization of the rumen NH_3_-N by ureolytic bacteria to synthesize MCP (evident by a numerically lower yield of MCP) or limited activity of urea transporters to facilitate NH_3_-N transport across the luminal and basolateral membrane of the rumen epithelium (Patra, 2015).

Furthermore, the analysis of rumen content microbial composition revealed that *Bacteroidetes* is the most abundant *Bacterial* phylum occupying 70.08% while *Prevotella* (52.15%) predominated the genera. This is slightly higher than those reported for Holstein cows by Wu et al. (2021) (*Bacteroidetes* = 51.4%, *Prevotella* = 38.48%) and Xue et al. (2020) (*Bacteroidetes* = 55.98%, *Prevotella* = 41.95%). This variation could be due to differences in the diet because the rumen of cows fed high concentrate diets are known to have a higher abundance of *Bacteroidetes* (Clemmons et al., 2019). At phylum levels, the relative abundances of all the four domains (*Bacteria*, *Archaea*, *Eukaryota*, and *Virus*) did not differ significantly between the HP and LP groups except for *Eukaryota* where *Euglenozoa* was significantly higher in the LP group (Supplementary Table 7 and Figure 2B). *Euglenozoa* are organisms with divergent roles which could either be autotrophic or obligate parasitism (Kostygov et al., 2021). In the *Archaea*, the genus *Methanothermobacter* was significantly higher in the HP group while *Thermoprotei_norank* was found to be significantly higher in the LP group (Supplementary Table 5 and Figure 2D). *Methanothermobacter* are hydrogenotrophic methanogens commonly found in anaerobic environments such as the rumen. They are known to synthesize enzymes, coenzymes, and prosthetic groups for methanogenesis (Bharathi and Chellapandi, 2017). There was no information regarding the activities of *Thermoprotei_norank* in the rumen. In the rumen *Bacteria*, the relative abundance of the genus *Sharpea* was significantly higher in the HP group when compared to the LP group (Supplementary Table 6 and Figure 2E). Likewise, Xue et al. (2019) reported a higher abundance of *Sharpea* in cows with high milk yield and milk protein yield when compared to the group with low milk yield. *Sharpea* is known to produce lactate, acetate, ethanol, and formate *via* their fermentation activities (Kumar et al., 2018). Interestingly, a higher relative abundance of *Sharpea* was reported in the rumen of sheep with low methane yield (Kamke et al., 2016). As for the *Eukaryota* genera, the HP group recorded a significantly higher abundance of *Trichomonas*, *Spironucleus*, *Allomyces*, *Saitoella*, and *Neospora* (Supplementary Table 7 and Figure 2A). *Spironucleus* were reported to cause diseases in a wide variety of vertebrates (Andersson et al., 2007; Millet et al., 2011) whereas *Trichomonas* sp. infects the rumen which affects the normal ruminal flora (Xu et al., 2021). A specie of *Neospora* (*Neospora caninum*) is known to cause abortion in cattle (De Marez et al., 1999). In the LP group, the relative abundance of *Kluyveromyces*, *Trypanosoma*, and *Bipolaris* were higher as compared to the HP group (Supplementary Table 7). *Trypanosoma* species usually inhabit the blood or intercellular fluid of their hosts and have been identified as the major cause of *trypanosomiasis* in cattle (Pathak, 2009). Although most of these *Eukaryota* genera are pathogenic or parasitic, the experimental cows were healthy and their milk production was not affected. Some species of *Kluyveromyces* (*Kluyveromyces lactis* and *K. marxianus*) were isolated from dairy products and were found to possess the gene (*LAC12–LAC4*) responsible for the breakdown of lactose (Lane et al., 2011). However, there is little information on the activities of *Kluyveromyces* in the rumen. Among the *Viruses*, *Siphoviridae_norank* was found to be significantly higher in the HP group while the LP group recorded a significantly higher abundance of *Teseptimavirus* (Supplementary Table 8 and Figure 2C). The *Siphoviridae* family are *bacteriophages* that mainly function to destroy various pathogenic microbiota including some strains of anti-biotic resistant *Salmonella* (Li et al., 2021).

In order to find out those microbiota that are associated with MP-metabolites and milk yield, we conducted a correlation analysis between the predominant microbiota and all the MP-metabolites. The results revealed that five *Bacteria* and two *Eukaryota* genera have a positive relationship with MP-metabolites. Among the *Bacteria*, *Ruminococcaceae_norank* is strongly associated with stearoylcarnitine and lysoPC (16:0) (serum) while *Butyrivibrio* has a positive relationship with allantoin (serum) and bilirubin (rumen). The genera *Lachnospiraceae_norank* and *Ruminococcus* both correlated with allantoin (serum). In the *Eukaryota*, both *Neocallimastix* and *Anaeromyces* have a positive association with 2-hydroxystearic acid (serum) (Figure 6 and Supplementary Table 12). *Ruminococcaceae* and *Lachnospiraceae* both specialize in the degradation of polysaccharides (Liang et al., 2021) while some species of *Butyrivibrio* (*B. fibrisolvens*) were reported to be involved in xylan fermentation and butyrate production (Emerson and Weimer, 2017). In the current study, correlation analysis revealed a strong and positive association between propionate percentage and some bacteria genera (*Ruminococcus*, *Eubacterium*, *Intestinibaculum*, and *Oribacterium*), signifying their role in the production of propionate. To corroborate this, *Eubacterium* was reported to be involved in propionate and formate synthesis (Zhou et al., 2015).

Furthermore, LefSe revealed that 15 CAZymes differ significantly between the HP and LP groups. In the HP group, the expression of CAZymes belonging to GH149, GT21, PL22, and PL5 were significantly enriched when compared to the LP group. However, the number of CAZymes significantly upregulated in the LP group (CBM56, CBM62, GH22, GH46, GT25, GT32, GT45, GT6, PL11, PL12, and PL27) were higher than those of the HP group (Supplementary Table 4 and Figure 3A). It is important to note that the CBMs (CBM56 and CBM62) were only enriched in the LP group. This is in line with the reports of Xue et al. (2020) and Wu et al. (2021) who recorded a higher number of genes encoding CBMs in low-producing cows. The CBMs are non-catalytic components within the CAZymes that target polysaccharides having D-galactose or L-arabinopyranose residues (Drula et al., 2022), hence suggesting a higher capacity of the cows with low milk yield in degrading complex polysaccharides. However, it is unclear exactly why the improved ability of the LP cows to degrade carbohydrates did not lead to a higher milk yield. Hence, further research is warranted.

The serum biochemistry showed that aspartate aminotransferase, albumin, and total cholesterol were found to be significantly higher in the HP when compared to the LP group (Table 4). The values of aspartate aminotransferase, albumin, and total cholesterol were within the ranges reported by Gu et al. (2021) in Chinese Holstein cows. Albumin helps in the maintenance of intravascular osmotic pressure while Cholesterol is an important precursor for the synthesis of some fat-soluble vitamins, steroid hormones, and bile acids (Washington and Van Hoosier, 2012).

In addition, metabolites identified by mass spectrometry were statistically analyzed and the results showed that 20 rumen metabolites, 24 milk metabolites, and 78 serum metabolites differed significantly between the HP and the LP group (Supplementary Table 9). The differential metabolites were matched to KEGG pathways and five pathways were significantly enriched in all the samples (two pathways in the milk, two pathways in the serum, and one pathway in the rumen fluid) (Supplementary Table 11). In the milk, arginine biosynthesis was significantly upregulated in the HP group while tyrosine metabolism was enriched in the LP group. Similarly, tyrosine metabolism was the only pathway upregulated in the LP group of the rumen fluid. In the serum, pyrimidine metabolism and beta-alanine metabolism were significantly enriched in the HP group while no pathway was enriched in the LP group. The metabolites that matched the KEGG pathways (Supplementary Table 11) include citrulline and *N*-acetylornithine (arginine biosynthesis in milk), dopamine, and gentisic acid (tyrosine metabolism in milk), as well as dopamine and 3-methoxytyramine (tyrosine metabolism in rumen fluid). In the serum, pyrimidine metabolism was impacted by five metabolites (uracil, cytidine, ureidopropionic acid, 2′-deoxyuridine, and uridine) while beta-alanine metabolism was matched to three serum metabolites (spermidine, ureidopropionic acid, and uracil). The upregulation of pyrimidine metabolism and beta-alanine metabolism in the serum of the HP group suggests an increased amino acid synthesis (beta-alanine) which could explain the increased milk protein yield in the HP group. Furthermore, it is worthy of note that tyrosine metabolism was significantly enriched in the LP group of both the rumen fluid and milk suggesting its negative association with milk yield.

**FIGURE 7:**
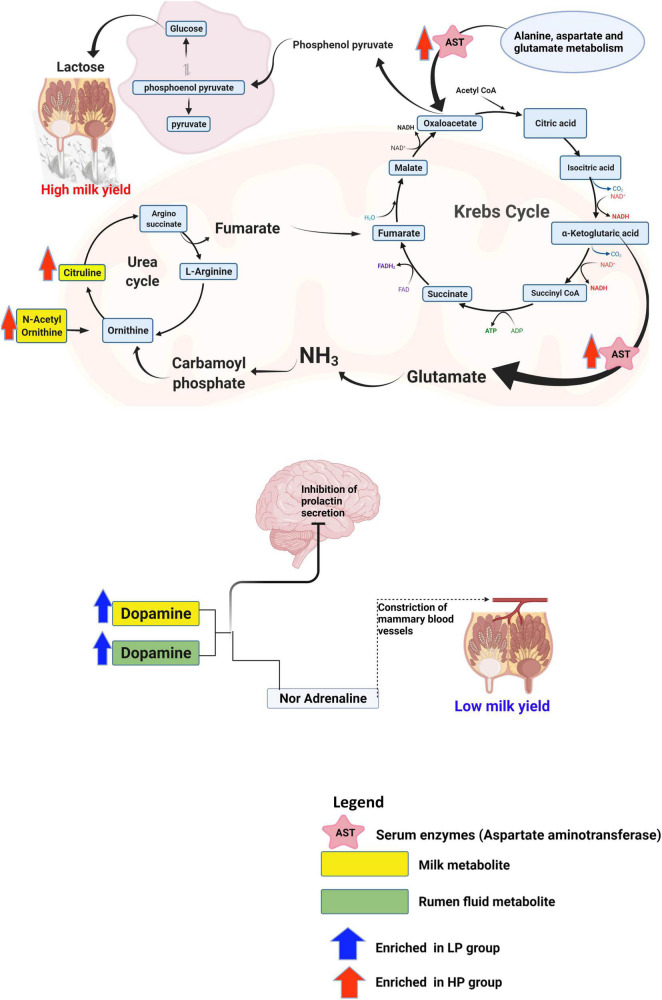
Schematics of the metabolic mechanism underlying the disparity in milk yield between the HP and LP groups. This figure was created using Biorender.com.

Therefore, to uncover the host’s metabolic mechanisms driving the disparity in milk yield between the HP and LP cows, we studied the metabolites that impacted tyrosine metabolism and arginine biosynthesis pathways. With regards to the metabolites that were matched to tyrosine metabolism, dopamine was identified to be significantly enriched in the rumen fluid and milk of the LP group (Supplementary Tables 11B,C). Dopamine is known to act on the type 2 dopamine receptors in the anterior pituitary to inhibit the release of prolactin (Grattan et al., 2008). Due to the key role of prolactin in the regulation of lactation (Lacasse and Ollier, 2015), reduced secretion of this hormone negatively affects lactation. Alternatively, dopamine could be converted to adrenaline and this hormone (adrenaline) negatively affects lactation. Adrenaline affects lactation either by narrowing the blood vessels and decreasing the flow of oxytocin to the mammary gland or directly inhibiting the action of oxytocin on myoepithelial cells (Bisset et al., 1967). Taken together, dopamine either acts on the anterior pituitary to inhibit the release of prolactin or it can be converted to adrenaline and eventually suppress the action of oxytocin in milk letdown (Figure 7). However, further research is needed to confirm the levels of adrenaline and prolactin in cows with low milk production in order to validate this mechanism. In the high-yielding cows (HP), we focused on the arginine biosynthesis pathway and its associated metabolites (citrulline, and *N*-acetylornithine) due to its role in supplying the substrates needed for energy metabolism. In the urea cycle, citrulline, and *N*-acetylornithine are converted to arginosuccinate and ornithine, respectively. The conversion of arginosuccinate to arginine produces fumarate which enters the citric acid cycle to be further converted to malate. Malate gets to the cytoplasm where it is oxidized to produce oxaloacetate. Alternatively, the aspartate aminotransferase (EC: 2.6.1.1), which was significantly enriched in the serum of the HP group, specializes in catalyzing the conversion of aspartate to oxaloacetate (Washington and Van Hoosier, 2012). In the mitochondrion, oxaloacetate is decarboxylated to form phosphoenolpyruvate and eventually converted to glucose *via* the gluconeogenic pathway (Figure 7). The free glucose can then be transported from the liver to the blood (Hanson and Owen, 2013) until it gets into the epithelial cells of the mammary glands, where it is either utilized for energy production or used for the synthesis of lactose (Nafikov and Beitz, 2007; Lin et al., 2016). In an *in vitro* study (Lin et al., 2016) on the mammary gland epithelial cells of cows, the infusion of glucose caused an increased lactose concentration and enhanced the expression of genes involved in glucose transportation and lactose biosynthesis pathway. Since lactose is the main the major regulator of milk yield in dairy cows (Lin et al., 2016), an increased yield of lactose will result in a higher milk yield.

## Conclusion

The study was able to identify some rumen microbiota, metabolites as well as metabolic pathways underlying the disparity in milk yield of Holstein cows. Rumen metagenomics revealed that two *Archaea*, one *Bacteria*, eight *Eukaryota*, and two *Viruses* differ between the HP and LP groups. In addition, the analysis of CAZymes showed that the CBMs, which are known to degrade polysaccharides, were only found in the LP group. The analysis of metabolites (rumen fluid, milk, and serum) obtained *via* mass spectrometry metabolomics revealed several differential metabolites between the HP and LP groups. Pathway enrichment analysis of the differential metabolites showed a total of five pathways significantly enriched in all the samples (two pathways in the milk, two pathways in the serum, and one pathway in the rumen fluid). Further investigation revealed that dopamine which was upregulated in the milk and rumen fluid of the LP group could be responsible for the low milk yield. Dopamine is known to inhibit the release of prolactin or suppress the action of oxytocin in the udder thereby resulting in reduced milk yield. On the other hand, the high milk yield in the HP group is attributed to an upregulation in citrulline, and *N*-acetylornithine, which are further metabolized *via* a series of reactions to produce oxaloacetate (a precursor of phosphoenolpyruvate). In addition, the aspartate aminotransferase, which was enriched in the serum of the HP group, specializes in catalyzing the conversion of aspartate to oxaloacetate. Further conversion of oxaloacetate produces phosphoenolpyruvate which eventually gets transformed to lactose in the mammary gland, thereby increasing milk yield.

## Data Availability Statement

The datasets presented in this study can be found in online repositories. The names of the repository/repositories and accession number(s) can be found in the article/Supplementary Material.

## Ethics Statement

The animal study was reviewed and approved by the ethics committee in strict adherence to the Animal Protection Law as stipulated in the Guide for the Care and Use of Laboratory Animals and approved by of Nanjing Agricultural University, Nanjing, China.

## Author Contributions

SM secured the funding, designed the experiment, supervised the project, and reviewed the manuscript. AA conducted the animal experiment work, performed the laboratory analysis, carried out the statistical analysis, and wrote the manuscript. LZ conducted the animal experiment work, performed the laboratory analysis and data analysis, and reviewed the manuscript. JZ organized the field work, conducted the data collection, and performed the laboratory analysis. All authors contributed to the article and approved the submitted version.

## Conflict of Interest

The authors declare that the research was conducted in the absence of any commercial or financial relationships that could be construed as a potential conflict of interest.

## Publisher’s Note

All claims expressed in this article are solely those of the authors and do not necessarily represent those of their affiliated organizations, or those of the publisher, the editors and the reviewers. Any product that may be evaluated in this article, or claim that may be made by its manufacturer, is not guaranteed or endorsed by the publisher.

## Footnotes

1
http://www.usadellab.org/cms/index.php?page=trimmomatic

2
http://bio-bwa.sourceforge.net/bwa.shtml

3
https://ccb.jhu.edu/software/bracken/

4
https://www.genome.jp/kegg/

5
http://kobas.cbi.pku.edu.cn/home.do

6
https://www.ncbi.nlm.nih.gov/sra/PRJNA748874

7
https://www.metaboanalyst.ca/

8
https://huttenhower.sph.harvard.edu/lefse/
