# Supplementary material for: Metagenomic and Metabolomic Insights Into the Mechanism Underlying the Disparity in Milk Yield of Holstein Cows
Source: Front Microbiol. 2022 May 20;13:844968. doi: 10.3389/fmicb.2022.844968 (PMC9163737; doi:10.3389/fmicb.2022.844968)
Supplement: Supplementary file 1 [file Table_1.DOCX]

### Table S1: Mobile phase elution procedure

| **Time (min)** | **Flow rate (mL/min)** | **A (%)** | **B (%)** |
| --- | --- | --- | --- |
| 0 | 0.3 | 95 | 5 |
| 1 | 0.3 | 95 | 5 |
| 2 | 0.3 | 60 | 40 |
| 7 | 0.3 | 20 | 80 |
| 11 | 0.3 | 5 | 95 |
| 15.5 | 0.3 | 95 | 5 |
| 19.5 | 0.3 | 95 | 5 |
