# Supplementary material for: Metagenomic and Metabolomic Insights Into the Mechanism Underlying the Disparity in Milk Yield of Holstein Cows
Source: Front Microbiol. 2022 May 20;13:844968. doi: 10.3389/fmicb.2022.844968 (PMC9163737; doi:10.3389/fmicb.2022.844968)
Supplement: Supplementary file 4 [file Table_4.DOCX]

**Table S4. Differential CAZymes in HP and LP groups**

| **Enzyme** | **Group enriched** | ***P*-value** | **LDA** |
| --- | --- | --- | --- |
| GH149 | HP | 0.038 | 2.81 |
| GT21 | HP | 0.047 | 2.69 |
| PL22 | HP | 0.047 | 2.61 |
| PL5 | HP | 0.024 | 2.87 |
| CBM56 | LP | 0.024 | 2.70 |
| CBM62 | LP | 0.002 | 2.84 |
| GH22 | LP | 0.015 | 3.14 |
| GH46 | LP | 0.019 | 2.77 |
| GT25 | LP | 0.015 | 2.69 |
| GT32 | LP | 0.024 | 2.60 |
| GT45 | LP | 0.047 | 2.93 |
| GT6 | LP | 0.012 | 3.01 |
| PL11 | LP | 0.047 | 2.66 |
| PL12 | LP | 0.047 | 2.80 |
| PL27 | LP | 0.047 | 2.83 |
