# Supplementary material for: Metagenomic and Metabolomic Insights Into the Mechanism Underlying the Disparity in Milk Yield of Holstein Cows
Source: Front Microbiol. 2022 May 20;13:844968. doi: 10.3389/fmicb.2022.844968 (PMC9163737; doi:10.3389/fmicb.2022.844968)
Supplement: Supplementary file 5 [file Table_5.DOCX]

**Table S5: Relative abundance (%) of predominant (>0.1 % in at least 1 sample) ruminal *Archaea***

**a) Phyla**

| **Phyla** | **HP** | **LP** | **SEM** | ***P*-value** | **LDA Score** |
| --- | --- | --- | --- | --- | --- |
| *Euryarchaeota* | 96.74 | 96.68 | 0.25 | 0.757 | NA |
| *Archaea_norank* | 1.94 | 1.95 | 0.17 | 0.895 | NA |
| *Candidatus Woesearchaeota* | 0.29 | 0.31 | 0.03 | 0.691 | NA |
| *Crenarchaeota* | 0.24 | 0.31 | 0.03 | 0.171 | NA |
| *Candidatus Bathyarchaeota* | 0.19 | 0.15 | 0.01 | 0.354 | NA |
| *Candidatus Thorarchaeota* | 0.12 | 0.13 | 0.02 | 0.757 | NA |
| *Candidatus Lokiarchaeota* | 0.11 | 0.12 | 0.01 | 0.895 | NA |
| *Thaumarchaeota* | 0.09 | 0.07 | 0.01 | 0.627 | NA |
| *Candidatus Aenigmarchaeota* | 0.07 | 0.05 | 0.01 | 0.825 | NA |
| *Nanoarchaeota* | 0.06 | 0.08 | 0.01 | 0.627 | NA |
| *Candidatus Altiarchaeota* | 0.04 | 0.05 | 0.01 | 0.825 | NA |
| *Candidatus Heimdallarchaeota* | 0.04 | 0.03 | 0.01 | 0.453 | NA |
| *Candidatus Micrarchaeota* | 0.01 | 0.02 | 0.01 | 0.825 | NA |

**b) Genera**

| **Genera** | **HP** | **LP** | **SEM** | ***P*-value** | **LDA Score** |
| --- | --- | --- | --- | --- | --- |
| *Methanobrevibacter* | 83.70 | 82.02 | 1.12 | 0.566 | NA |
| *Methanosarcina* | 3.41 | 3.87 | 0.30 | 0.566 | NA |
| *Methanosphaera* | 2.39 | 2.44 | 0.11 | 0.895 | NA |
| *Thermoplasmata_norank* | 2.05 | 2.21 | 0.29 | 0.965 | NA |
| *Archaea_norank* | 1.94 | 1.95 | 0.17 | 0.895 | NA |
| *Methanomicrobia_norank* | 0.66 | 1.10 | 0.20 | 0.31 | NA |
| *Methanomassiliicoccus* | 0.57 | 0.69 | 0.10 | 0.825 | NA |
| *Methanobacterium* | 0.56 | 0.53 | 0.03 | 0.627 | NA |
| *Methanomassiliicoccales_norank* | 0.47 | 0.72 | 0.07 | 0.122 | NA |
| *Candidatus Woesearchaeota_norank* | 0.29 | 0.31 | 0.03 | 0.691 | NA |
| *Euryarchaeota_norank* | 0.27 | 0.28 | 0.02 | 0.895 | NA |
| *Methanomicrobium* | 0.21 | 0.21 | 0.03 | 0.627 | NA |
| *Candidatus Bathyarchaeota_norank* | 0.19 | 0.15 | 0.01 | 0.354 | NA |
| *Thermoplasmatales_norank* | 0.18 | 0.15 | 0.02 | 0.965 | NA |
| *Methanobacteriales_norank* | 0.18 | 0.24 | 0.02 | 0.2 | NA |
| *Candidatus Methanomethylophilus* | 0.18 | 0.14 | 0.03 | 0.691 | NA |
| *Methanolobus* | 0.16 | 0.19 | 0.04 | 0.895 | NA |
| *Methanoculleus* | 0.16 | 0.15 | 0.02 | 0.627 | NA |
| *Methanosarcinales_norank* | 0.16 | 0.16 | 0.02 | 0.627 | NA |
| *Thermococcus* | 0.14 | 0.14 | 0.01 | 0.691 | NA |
| *Thermoprotei_norank* | 0.14 | 0.22 | 0.03 | 0.047 | 2.08 |
| *Candidatus Thorarchaeota_norank* | 0.12 | 0.13 | 0.02 | 0.757 | NA |
| *Archaeoglobales_norank* | 0.12 | 0.11 | 0.01 | 0.757 | NA |
| *Candidatus Lokiarchaeota_norank* | 0.11 | 0.11 | 0.01 | 0.825 | NA |
| *Methanothermobacter* | 0.10 | 0.05 | 0.01 | 0.012 | 2.34 |
| *Methanocorpusculum* | 0.09 | 0.11 | 0.01 | 0.31 | NA |
| *Methanocaldococcus* | 0.08 | 0.09 | 0.01 | 0.965 | NA |
| *Methanomassiliicoccaceae_norank* | 0.08 | 0.05 | 0.02 | 0.757 | NA |
| *Haloferax* | 0.07 | 0.04 | 0.02 | 0.566 | NA |
| *Methanohalophilus* | 0.07 | 0.09 | 0.02 | 0.895 | NA |
| *Candidatus Aenigmarchaeota_norank* | 0.07 | 0.05 | 0.01 | 0.825 | NA |
| *Thermococci_norank* | 0.07 | 0.07 | 0.01 | 0.453 | NA |
| *Nanoarchaeota_norank* | 0.06 | 0.08 | 0.01 | 0.965 | NA |
| *Methanococcoides* | 0.05 | 0.04 | 0.01 | 0.965 | NA |
| *Candidatus Methanofastidiosum* | 0.04 | 0.06 | 0.01 | 0.058 | NA |
| *Candidatus Heimdallarchaeota_norank* | 0.04 | 0.03 | 0.01 | 0.453 | NA |
| *Nitrosopumilaceae_norank* | 0.03 | 0.01 | 0.01 | 0.691 | NA |
| *Methanobacteriales_norank* | 0.03 | 0.04 | 0.01 | 0.627 | NA |
| *Caldisphaera* | 0.03 | 0.00 | 0.01 | 0.423 | NA |
| *Theionarchaea_norank* | 0.03 | 0.05 | 0.01 | 0.171 | NA |
| *Halorubrum* | 0.02 | 0.05 | 0.01 | 0.757 | NA |
| *Methanohalobium* | 0.01 | 0.03 | 0.01 | 0.058 | NA |
| *Candidatus Micrarchaeota_norank* | 0.01 | 0.02 | 0.01 | 0.825 | NA |
| *Natronolimnobius* | 0.00 | 0.02 | 0.01 | 0.393 | NA |
| *Thermofilum* | 0.00 | 0.02 | 0.01 | 0.923 | NA |
| *Natronorubrum* | 0.00 | 0.02 | 0.01 | 0.696 | NA |

NA: Not Applicable.

Only Phyla and genera with LDA Score >2 are displayed.
