# Supplementary material for: Metagenomic and Metabolomic Insights Into the Mechanism Underlying the Disparity in Milk Yield of Holstein Cows
Source: Front Microbiol. 2022 May 20;13:844968. doi: 10.3389/fmicb.2022.844968 (PMC9163737; doi:10.3389/fmicb.2022.844968)
Supplement: Supplementary file 6 [file Table_6.DOCX]

**Table S6: Relative abundance (%) of predominant (>0.1 % in at least 1 sample) ruminal *Bacteria***

**a) Phyla**

| **Phyla** | **HP** | **LP** | **SEM** | ***P*-value** | **LDA Score** |
| --- | --- | --- | --- | --- | --- |
| *Bacteroidetes* | 68.34 | 71.82 | 1.81 | 0.626 | NA |
| *Firmicutes* | 21.43 | 17.73 | 1.32 | 0.251 | NA |
| *Bacteria_norank* | 3.21 | 3.55 | 0.21 | 0.453 | NA |
| *Proteobacteria* | 3.01 | 3.30 | 0.56 | 0.627 | NA |
| *Fibrobacteres* | 1.68 | 1.46 | 0.21 | 0.627 | NA |
| *Actinobacteria* | 0.94 | 0.69 | 0.18 | 0.757 | NA |
| *Spirochaetes* | 0.43 | 0.42 | 0.02 | 0.757 | NA |
| *Verrucomicrobia* | 0.26 | 0.33 | 0.06 | 0.627 | NA |
| *Cyanobacteria* | 0.09 | 0.08 | 0.02 | 0.757 | NA |
| *Candidatus Saccharibacteria* | 0.09 | 0.09 | 0.01 | 0.965 | NA |
| *Lentisphaerae* | 0.06 | 0.08 | 0.01 | 0.757 | NA |
| *Candidatus Melainabacteria* | 0.06 | 0.05 | 0.01 | 0.965 | NA |
| *Tenericutes* | 0.05 | 0.05 | 0.01 | 0.401 | NA |
| *Planctomycetes* | 0.05 | 0.06 | 0.01 | 0.965 | NA |
| *Elusimicrobia* | 0.02 | 0.01 | 0.01 | 0.289 | NA |

**b) Genera**

| **Genera** | **HP** | **LP** | **SEM** | ***P*-value** | **LDA Score** |
| --- | --- | --- | --- | --- | --- |
| *Prevotella* | 50.63 | 53.67 | 1.95 | 0.566 | NA |
| *Prevotellaceae_norank* | 5.31 | 6.29 | 0.51 | 0.895 | NA |
| *Bacteroides* | 3.66 | 3.58 | 0.08 | 0.453 | NA |
| *Bacteria_norank* | 3.21 | 3.55 | 0.21 | 0.453 | NA |
| *Lachnospiraceae_norank* | 4.03 | 3.26 | 0.39 | 0.27 | NA |
| *Bacteroidales_norank* | 2.77 | 2.66 | 0.15 | 0.895 | NA |
| *Clostridiales_norank* | 2.03 | 1.91 | 0.15 | 0.627 | NA |
| *Ruminococcus* | 2.40 | 1.74 | 0.23 | 0.145 | NA |
| *Fibrobacter* | 1.68 | 1.46 | 0.21 | 0.627 | NA |
| *Ruminococcaceae_norank* | 1.78 | 1.25 | 0.13 | 0.058 | NA |
| *Rikenellaceae_norank* | 1.14 | 1.20 | 0.10 | 0.691 | NA |
| *Selenomonas* | 1.15 | 1.09 | 0.10 | 0.757 | NA |
| *Porphyromonadaceae_norank* | 1.45 | 1.04 | 0.16 | 0.31 | NA |
| *Succiniclasticum* | 0.78 | 0.93 | 0.11 | 0.757 | NA |
| *Butyrivibrio* | 0.89 | 0.81 | 0.04 | 0.27 | NA |
| *Clostridium* | 0.76 | 0.62 | 0.07 | 0.566 | NA |
| *Eubacterium* | 0.82 | 0.56 | 0.11 | 0.2 | NA |
| *Alistipes* | 0.59 | 0.52 | 0.03 | 0.402 | NA |
| *Firmicutes_norank* | 0.65 | 0.51 | 0.05 | 0.122 | NA |
| *Parabacteroides* | 0.37 | 0.38 | 0.01 | 0.453 | NA |
| *Oribacterium* | 0.35 | 0.38 | 0.06 | 0.965 | NA |
| *Clostridia_norank* | 0.38 | 0.38 | 0.03 | 0.965 | NA |
| *Lachnoclostridium* | 0.36 | 0.31 | 0.03 | 0.354 | NA |
| *Alloprevotella* | 0.32 | 0.30 | 0.01 | 0.402 | NA |
| *Treponema* | 0.31 | 0.29 | 0.02 | 0.453 | NA |
| *Aeromonas* | 0.27 | 0.28 | 0.06 | 0.402 | NA |
| *Verrucomicrobia_norank* | 0.20 | 0.26 | 0.05 | 0.627 | NA |
| *Ruminobacter* | 0.22 | 0.25 | 0.04 | 0.895 | NA |
| *Sarcina* | 0.26 | 0.22 | 0.02 | 0.31 | NA |
| *Pseudoscardovia* | 0.48 | 0.22 | 0.14 | 0.566 | NA |
| *Succinimonas* | 0.20 | 0.22 | 0.04 | 0.825 | NA |
| *Bacteroidetes_norank* | 0.18 | 0.21 | 0.02 | 0.965 | NA |
| *Erysipelotrichaceae_norank* | 0.35 | 0.21 | 0.06 | 0.233 | NA |
| *Blautia* | 0.24 | 0.19 | 0.02 | 0.31 | NA |
| *Succinivibrionaceae_norank* | 0.17 | 0.19 | 0.04 | 0.757 | NA |
| *Succinivibrio* | 0.15 | 0.17 | 0.03 | 0.757 | NA |
| *Muribaculaceae_norank* | 0.19 | 0.17 | 0.01 | 0.31 | NA |
| *Oscillibacter* | 0.22 | 0.17 | 0.02 | 0.171 | NA |
| *Tolumonas* | 0.16 | 0.16 | 0.04 | 0.453 | NA |
| *Roseburia* | 0.22 | 0.16 | 0.03 | 0.233 | NA |
| *Bacteroidia_norank* | 0.21 | 0.16 | 0.04 | 0.566 | NA |
| *Pseudobutyrivibrio* | 0.18 | 0.15 | 0.01 | 0.233 | NA |
| *Paraprevotella* | 0.14 | 0.13 | 0.01 | 0.402 | NA |
| *Succinatimonas* | 0.12 | 0.13 | 0.03 | 0.453 | NA |
| *Faecalibacterium* | 0.13 | 0.12 | 0.01 | 0.566 | NA |
| *Schwartzia* | 0.12 | 0.11 | 0.01 | 0.453 | NA |
| *Lachnospira* | 0.15 | 0.10 | 0.02 | 0.354 | NA |
| *Paludibacter* | 0.07 | 0.10 | 0.01 | 0.171 | NA |
| *Bifidobacterium* | 0.08 | 0.10 | 0.02 | 0.402 | NA |
| *Olsenella* | 0.11 | 0.10 | 0.01 | 0.453 | NA |
| *Barnesiella* | 0.10 | 0.10 | 0.01 | 0.825 | NA |
| *Megasphaera* | 0.10 | 0.09 | 0.03 | 0.895 | NA |
| *Vibrio* | 0.09 | 0.09 | 0.02 | 0.453 | NA |
| *Escherichia* | 0.08 | 0.09 | 0.01 | 0.895 | NA |
| *Intestinibaculum* | 0.27 | 0.09 | 0.10 | 0.171 | NA |
| *Gammaproteobacteria_norank* | 0.08 | 0.09 | 0.02 | 0.691 | NA |
| *Candidatus Saccharibacteria_norank* | 0.09 | 0.08 | 0.01 | 0.965 | NA |
| *Sutterella* | 0.06 | 0.08 | 0.01 | 0.233 | NA |
| *Dialister* | 0.12 | 0.08 | 0.03 | 0.627 | NA |
| *Flavonifractor* | 0.09 | 0.08 | 0.01 | 0.627 | NA |
| *Acidaminococcaceae_norank* | 0.07 | 0.08 | 0.02 | 0.965 | NA |
| *Salmonella* | 0.06 | 0.07 | 0.01 | 0.965 | NA |
| *Lactobacillus* | 0.09 | 0.07 | 0.01 | 0.757 | NA |
| *Anaerovibrio* | 0.06 | 0.07 | 0.01 | 0.965 | NA |
| *Cyanobacteria_norank* | 0.07 | 0.07 | 0.02 | 0.757 | NA |
| *Pseudoflavonifractor* | 0.07 | 0.06 | 0.01 | 0.757 | NA |
| *Clostridiaceae_norank* | 0.07 | 0.06 | 0.00 | 0.453 | NA |
| *Acidaminococcus* | 0.06 | 0.05 | 0.02 | 0.691 | NA |
| *Pseudomonas* | 0.04 | 0.05 | 0.01 | 0.895 | NA |
| *Coprococcus* | 0.06 | 0.05 | 0.01 | 0.31 | NA |
| *Agathobacter* | 0.06 | 0.05 | 0.01 | 0.145 | NA |
| *Mitsuokella* | 0.04 | 0.05 | 0.01 | 0.757 | NA |
| *Alphaproteobacteria_norank* | 0.06 | 0.05 | 0.01 | 0.508 | NA |
| *Sphingobacteriia_norank* | 0.03 | 0.05 | 0.00 | 0.085 | NA |
| *Dorea* | 0.06 | 0.05 | 0.00 | 0.453 | NA |
| *Ralstonia* | 0.03 | 0.04 | 0.01 | 0.508 | NA |
| *Shewanella* | 0.03 | 0.03 | 0.01 | 0.453 | NA |
| *Lactimicrobium* | 0.03 | 0.03 | 0.01 | 0.825 | NA |
| *Eubacteriaceae_norank* | 0.04 | 0.03 | 0.01 | 0.171 | NA |
| *Anaerolactibacter* | 0.03 | 0.02 | 0.01 | 0.31 | NA |
| *Sharpea* | 0.04 | 0.02 | 0.01 | 0.047 | 2.14 |
| *Kandleria* | 0.03 | 0.01 | 0.01 | 0.402 | NA |

NA: Not Applicable.

Only Phyla and genera with LDA Score >2 are displayed.
