# Supplementary material for: Metagenomic and Metabolomic Insights Into the Mechanism Underlying the Disparity in Milk Yield of Holstein Cows
Source: Front Microbiol. 2022 May 20;13:844968. doi: 10.3389/fmicb.2022.844968 (PMC9163737; doi:10.3389/fmicb.2022.844968)
Supplement: Supplementary file 7 [file Table_7.DOCX]

**Table S7: Relative abundance (%) of predominant (>0.1 % in at least 1 sample) ruminal *Eukaryota***

**a) Phyla**

| **Phyla** | **HP** | **LP** | **SEM** | ***P*-value** | **LDA Score** |
| --- | --- | --- | --- | --- | --- |
| *Ciliophora* | 63.16 | 62.57 | 0.83 | 0.627 | NA |
| *Chytridiomycota* | 8.74 | 8.18 | 0.51 | 0.965 | NA |
| *Eukaryota_norank* | 5.88 | 5.88 | 0.06 | 0.965 | NA |
| *Ascomycota* | 4.20 | 5.57 | 0.44 | 0.171 | NA |
| *Apicomplexa* | 3.14 | 2.98 | 0.06 | 0.122 | NA |
| *Evosea* | 2.34 | 2.45 | 0.04 | 0.233 | NA |
| *Mucoromycota* | 1.47 | 1.55 | 0.04 | 0.691 | NA |
| *Parabasalia* | 1.46 | 1.51 | 0.04 | 0.825 | NA |
| *Basidiomycota* | 1.12 | 1.10 | 0.02 | 0.965 | NA |
| *Heterolobosea* | 1.03 | 1.03 | 0.03 | 0.965 | NA |
| *Preaxostyla* | 0.90 | 0.71 | 0.21 | 0.757 | NA |
| *Zoopagomycota* | 0.75 | 0.73 | 0.02 | 0.508 | NA |
| *Platyhelminthes* | 0.73 | 0.68 | 0.02 | 0.058 | NA |
| *Rhodophyta* | 0.55 | 0.53 | 0.01 | 0.354 | NA |
| *Perkinsozoa* | 0.45 | 0.41 | 0.01 | 0.122 | NA |
| *Discosea* | 0.41 | 0.42 | 0.07 | 0.691 | NA |
| *Endomyxa* | 0.34 | 0.34 | 0.02 | 0.825 | NA |
| *Bacillariophyta* | 0.31 | 0.31 | 0.02 | 0.895 | NA |
| *Echinodermata* | 0.31 | 0.29 | 0.01 | 0.122 | NA |
| *Euglenozoa* | 0.29 | 0.38 | 0.03 | 0.015 | 2.21 |
| *Blastocladiomycota* | 0.27 | 0.23 | 0.01 | 0.171 | NA |
| *Rotifera* | 0.24 | 0.24 | 0.01 | 0.825 | NA |
| *Porifera* | 0.24 | 0.22 | 0.01 | 0.200 | NA |
| *Annelida* | 0.24 | 0.22 | 0.01 | 0.270 | NA |
| *Fornicata* | 0.23 | 0.20 | 0.01 | 0.122 | NA |
| *Microsporidia* | 0.20 | 0.21 | 0.01 | 0.627 | NA |
| *Cryptomycota* | 0.18 | 0.19 | 0.01 | 0.354 | NA |
| *Foraminifera* | 0.13 | 0.13 | 0.00 | 0.627 | NA |
| *Placozoa* | 0.13 | 0.14 | 0.00 | 0.270 | NA |
| *Brachiopoda* | 0.13 | 0.13 | 0.00 | 0.757 | NA |
| *Haptista* | 0.08 | 0.09 | 0.01 | 0.270 | NA |
| *Priapulida* | 0.08 | 0.06 | 0.00 | 0.145 | NA |
| *Nemertea* | 0.07 | 0.05 | 0.01 | 0.233 | NA |
| *Tardigrada* | 0.07 | 0.08 | 0.01 | 0.200 | NA |

**b) Genera**

| **Genera** | **HP** | **LP** | **SEM** | ***P*-value** | **LDA Score** |
| --- | --- | --- | --- | --- | --- |
| *Stylonychia* | 16.46 | 16.58 | 0.27 | 0.825 | NA |
| *Stentor* | 10.49 | 10.45 | 0.14 | 0.627 | NA |
| *Tetrahymena* | 8.39 | 8.28 | 0.14 | 0.825 | NA |
| *Paramecium* | 7.18 | 7.27 | 0.10 | 0.453 | NA |
| *Halteria* | 6.00 | 6.04 | 0.08 | 0.627 | NA |
| *Pseudocohnilembus* | 4.82 | 4.90 | 0.06 | 0.402 | NA |
| *Ichthyophthirius* | 4.61 | 4.59 | 0.07 | 0.757 | NA |
| *Piromyces* | 3.75 | 3.58 | 0.37 | 0.965 | NA |
| *Entodinium* | 3.49 | 2.73 | 0.17 | 0.085 | NA |
| *Neocallimastix* | 3.04 | 2.86 | 0.11 | 0.31 | NA |
| *Anaeromyces* | 1.23 | 1.06 | 0.06 | 0.233 | NA |
| *Plasmodium* | 1.17 | 1.08 | 0.02 | 0.085 | NA |
| *Vitrella* | 1.10 | 1.12 | 0.02 | 0.566 | NA |
| *Naegleria* | 1.03 | 1.03 | 0.03 | 0.965 | NA |
| *Streblomastix* | 0.90 | 0.70 | 0.21 | 0.895 | NA |
| *Trichomonas* | 0.74 | 0.66 | 0.02 | 0.047 | 2.01 |
| *Tritrichomonas* | 0.72 | 0.85 | 0.04 | 0.102 | NA |
| *Monosporascus* | 0.68 | 0.91 | 0.20 | 0.965 | NA |
| *Entamoeba* | 0.63 | 0.64 | 0.02 | 0.757 | NA |
| *Babesia* | 0.49 | 0.40 | 0.03 | 0.07 | NA |
| *Dictyostelium* | 0.48 | 0.50 | 0.02 | 0.453 | NA |
| *Pythium* | 0.46 | 0.42 | 0.02 | 0.825 | NA |
| *Perkinsus* | 0.45 | 0.41 | 0.01 | 0.122 | NA |
| *Blastocystis* | 0.44 | 0.43 | 0.01 | 0.627 | NA |
| *Euplotoides* | 0.42 | 0.41 | 0.01 | 0.965 | NA |
| *Guillardia* | 0.41 | 0.40 | 0.02 | 0.566 | NA |
| *Acanthamoeba* | 0.41 | 0.42 | 0.07 | 0.691 | NA |
| *Aphanomyces* | 0.39 | 0.39 | 0.01 | 0.895 | NA |
| *Cryptosporidium* | 0.37 | 0.38 | 0.01 | 0.691 | NA |
| *Plasmodiophora* | 0.34 | 0.34 | 0.02 | 0.825 | NA |
| *Albugo* | 0.33 | 0.33 | 0.01 | 0.825 | NA |
| *Theileria* | 0.32 | 0.33 | 0.01 | 0.354 | NA |
| *Phytophthora* | 0.31 | 0.32 | 0.01 | 0.627 | NA |
| *Acytostelium* | 0.30 | 0.32 | 0.01 | 0.233 | NA |
| *Galdieria* | 0.27 | 0.22 | 0.02 | 0.058 | NA |
| *Basidiobolus* | 0.27 | 0.23 | 0.01 | 0.2 | NA |
| *Monosiga* | 0.26 | 0.25 | 0.01 | 0.691 | NA |
| *Lichtheimia* | 0.26 | 0.26 | 0.01 | 0.965 | NA |
| *Rhizophagus* | 0.25 | 0.28 | 0.02 | 0.233 | NA |
| *Planoprotostelium* | 0.25 | 0.26 | 0.01 | 0.508 | NA |
| *Brachionus* | 0.24 | 0.23 | 0.01 | 0.895 | NA |
| *Hanseniaspora* | 0.23 | 0.20 | 0.01 | 0.07 | NA |
| *Symbiodinium* | 0.23 | 0.25 | 0.01 | 0.27 | NA |
| *Rhizopus* | 0.23 | 0.24 | 0.01 | 0.965 | NA |
| *Amphimedon* | 0.22 | 0.19 | 0.01 | 0.058 | NA |
| *Polysphondylium* | 0.22 | 0.22 | 0.01 | 0.825 | NA |
| *Batrachochytrium* | 0.20 | 0.16 | 0.02 | 0.2 | NA |
| *Spironucleus* | 0.19 | 0.17 | 0.01 | 0.038 | 2.37 |
| *Isotricha* | 0.19 | 0.20 | 0.01 | 0.31 | NA |
| *Thraustotheca* | 0.18 | 0.20 | 0.01 | 0.691 | NA |
| *Heterostelium* | 0.17 | 0.18 | 0.01 | 0.825 | NA |
| *Smittium* | 0.17 | 0.17 | 0.01 | 0.965 | NA |
| *Allomyces* | 0.17 | 0.14 | 0.01 | 0.019 | 2.33 |
| *Helobdella* | 0.17 | 0.16 | 0.01 | 0.145 | NA |
| *Synchytrium* | 0.17 | 0.13 | 0.01 | 0.354 | NA |
| *Acanthaster* | 0.16 | 0.14 | 0.01 | 0.31 | NA |
| *Eudiplodinium* | 0.16 | 0.15 | 0.01 | 0.627 | NA |
| *Hondaea* | 0.16 | 0.18 | 0.01 | 0.2 | NA |
| *Sterkiella* | 0.16 | 0.17 | 0.01 | 0.453 | NA |
| *Tieghemostelium* | 0.15 | 0.17 | 0.01 | 0.354 | NA |
| *Neospora* | 0.15 | 0.12 | 0.01 | 0.005 | 2.41 |
| *Cryptocaryon* | 0.15 | 0.15 | 0.01 | 0.965 | NA |
| *Gracilariopsis* | 0.14 | 0.16 | 0.01 | 0.27 | NA |
| *Clonorchis* | 0.14 | 0.07 | 0.02 | 0.009 | NA |
| *Cafeteria* | 0.14 | 0.14 | 0.01 | 0.453 | NA |
| *Thalassiosira* | 0.14 | 0.14 | 0.02 | 0.825 | NA |
| *Aspergillus* | 0.13 | 0.13 | 0.01 | 0.825 | NA |
| *Trichoplax* | 0.13 | 0.14 | 0.00 | 0.27 | NA |
| *Besnoitia* | 0.13 | 0.12 | 0.00 | 0.171 | NA |
| *Toxoplasma* | 0.13 | 0.11 | 0.01 | 0.233 | NA |
| *Cavenderia* | 0.13 | 0.14 | 0.00 | 0.085 | NA |
| *Euplotes* | 0.13 | 0.14 | 0.01 | 0.31 | NA |
| *Lingula* | 0.13 | 0.13 | 0.00 | 0.757 | NA |
| *Globisporangium* | 0.13 | 0.15 | 0.01 | 0.102 | NA |
| *Reticulomyxa* | 0.13 | 0.12 | 0.00 | 0.757 | NA |
| *Thecamonas* | 0.13 | 0.12 | 0.01 | 0.965 | NA |
| *Aureococcus* | 0.13 | 0.11 | 0.02 | 0.825 | NA |
| *Schizosaccharomyces* | 0.12 | 0.10 | 0.01 | 0.27 | NA |
| *Saprolegnia* | 0.12 | 0.13 | 0.00 | 0.233 | NA |
| *Rozella* | 0.12 | 0.13 | 0.01 | 0.402 | NA |
| *Andalucia* | 0.11 | 0.11 | 0.01 | 0.402 | NA |
| *Trypanosoma* | 0.11 | 0.19 | 0.02 | 0.007 | 2.06 |
| *Chaetomium* | 0.11 | 0.10 | 0.01 | 0.627 | NA |
| *Eimeria* | 0.11 | 0.12 | 0.01 | 0.145 | NA |
| *Moneuplotes* | 0.11 | 0.10 | 0.01 | 0.27 | NA |
| *Salpingoeca* | 0.11 | 0.09 | 0.01 | 0.27 | NA |
| *Capsaspora* | 0.10 | 0.11 | 0.00 | 0.233 | NA |
| *Achlya* | 0.10 | 0.09 | 0.00 | 0.691 | NA |
| *Conidiobolus* | 0.10 | 0.12 | 0.01 | 0.102 | NA |
| *Epidinium* | 0.10 | 0.06 | 0.01 | 0.27 | NA |
| *Strongylocentrotus* | 0.10 | 0.10 | 0.01 | 0.508 | NA |
| *Schistosoma* | 0.10 | 0.11 | 0.00 | 0.566 | NA |
| *Ogataea* | 0.10 | 0.10 | 0.00 | 0.757 | NA |
| *Catenaria* | 0.10 | 0.10 | 0.00 | 0.757 | NA |
| *Macrostomum* | 0.09 | 0.09 | 0.00 | 0.757 | NA |
| *Saccharomyces* | 0.09 | 0.08 | 0.01 | 0.895 | NA |
| *Wallemia* | 0.09 | 0.11 | 0.00 | 0.102 | NA |
| *Hepatocystis* | 0.09 | 0.10 | 0.01 | 0.27 | NA |
| *Plasmopara* | 0.09 | 0.08 | 0.00 | 0.627 | NA |
| *Ramularia* | 0.09 | 0.13 | 0.03 | 0.402 | NA |
| *Saitoella* | 0.08 | 0.05 | 0.01 | 0.003 | 2.44 |
| *Rhizoclosmatium* | 0.08 | 0.08 | 0.01 | 0.757 | NA |
| *Ciliophora_norank* | 0.08 | 0.06 | 0.01 | 0.453 | NA |
| *Hesseltinella* | 0.08 | 0.09 | 0.01 | 0.402 | NA |
| *Kazachstania* | 0.08 | 0.08 | 0.01 | 0.895 | NA |
| *Spizellomyces* | 0.08 | 0.08 | 0.00 | 0.508 | NA |
| *Priapulus* | 0.08 | 0.06 | 0.00 | 0.145 | NA |
| *Orpinomyces* | 0.08 | 0.07 | 0.02 | 0.825 | NA |
| *Pneumocystis* | 0.07 | 0.08 | 0.00 | 0.691 | NA |
| *Clavispora* | 0.07 | 0.06 | 0.01 | 0.31 | NA |
| *Gregarina* | 0.07 | 0.06 | 0.01 | 0.102 | NA |
| *Colletotrichum* | 0.07 | 0.08 | 0.03 | 0.31 | NA |
| *Coemansia* | 0.07 | 0.05 | 0.01 | 0.2 | NA |
| *Cerebratulus* | 0.07 | 0.05 | 0.01 | 0.233 | NA |
| *Choanephora* | 0.07 | 0.08 | 0.01 | 0.354 | NA |
| *Malassezia* | 0.06 | 0.05 | 0.01 | 0.171 | NA |
| *Brettanomyces* | 0.06 | 0.05 | 0.00 | 0.31 | NA |
| *Fusarium* | 0.06 | 0.07 | 0.01 | 0.691 | NA |
| *Diversispora* | 0.06 | 0.07 | 0.00 | 0.058 | NA |
| *Chrysochromulina* | 0.06 | 0.07 | 0.00 | 0.453 | NA |
| *Pyricularia* | 0.05 | 0.06 | 0.01 | 0.171 | NA |
| *Bodo* | 0.05 | 0.07 | 0.01 | 0.122 | NA |
| *Trichoderma* | 0.05 | 0.03 | 0.01 | 0.825 | NA |
| *Jimgerdemannia* | 0.04 | 0.03 | 0.01 | 0.627 | NA |
| *Giardia* | 0.04 | 0.03 | 0.01 | 0.965 | NA |
| *Metarhizium* | 0.02 | 0.04 | 0.01 | 0.895 | NA |
| *Tilletia* | 0.02 | 0.01 | 0.01 | 0.627 | NA |
| *Cliftonaea* | 0.02 | 0.03 | 0.01 | 0.387 | NA |
| *Kluyveromyces* | 0.02 | 1.08 | 0.29 | 0.047 | 2.92 |
| *Lentinula* | 0.02 | 0.04 | 0.01 | 0.31 | NA |
| *Ordospora* | 0.00 | 0.02 | 0.01 | 0.171 | NA |
| *Bipolaris* | 0.00 | 0.02 | 0.01 | 0.046 | 2.26 |

NA: Not Applicable.

Only Phyla and genera with LDA Score >2 are displayed.
