# Supplementary material for: Metagenomic and Metabolomic Insights Into the Mechanism Underlying the Disparity in Milk Yield of Holstein Cows
Source: Front Microbiol. 2022 May 20;13:844968. doi: 10.3389/fmicb.2022.844968 (PMC9163737; doi:10.3389/fmicb.2022.844968)
Supplement: Supplementary file 8 [file Table_8.DOCX]

**Table S8: Relative abundance (%) of predominant (>0.1 % in at least 1 sample) ruminal *Viruses***

| **Genera** | **HP** | **LP** | **SEM** | ***P*-value** | **LDA Score** |
| --- | --- | --- | --- | --- | --- |
| *Podoviridae_norank* | 37.77 | 36.46 | 1.88 | 0.825 | NA |
| *Siphoviridae_norank* | 26.68 | 21.49 | 1.10 | 0.031 | 3.48 |
| *Virus_norank* | 9.75 | 12.56 | 1.18 | 0.757 | NA |
| *Myoviridae_norank* | 6.75 | 8.13 | 0.61 | 0.2 | NA |
| *Cequinquevirus* | 5.48 | 6.10 | 1.25 | 0.566 | NA |
| *Ithacavirus* | 1.72 | 2.03 | 0.48 | 0.825 | NA |
| *Phikzvirus* | 1.57 | 2.04 | 0.41 | 0.825 | NA |
| *Caudovirales_norank* | 0.88 | 0.81 | 0.09 | 0.233 | NA |
| *Mimiviridae_norank* | 0.81 | 0.78 | 0.14 | 0.691 | NA |
| *Herelleviridae_norank* | 0.50 | 0.33 | 0.08 | 0.895 | NA |
| *Microviridae_norank* | 0.47 | 0.45 | 0.09 | 0.508 | NA |
| *Doucettevirus* | 0.38 | 0.28 | 0.12 | 0.965 | NA |
| *Noxifervirus* | 0.37 | 0.42 | 0.10 | 0.508 | NA |
| *Scapunavirus* | 0.35 | 0.28 | 0.02 | 0.145 | NA |
| *Chiangmaivirus* | 0.33 | 0.42 | 0.10 | 0.825 | NA |
| *Pithoviridae_norank* | 0.25 | 0.24 | 0.05 | 0.825 | NA |
| *Agricanvirus* | 0.24 | 0.40 | 0.09 | 0.895 | NA |
| *Hokovirus* | 0.21 | 0.22 | 0.05 | 0.825 | NA |
| *Gordonvirus* | 0.19 | 0.15 | 0.02 | 0.453 | NA |
| *Machinavirus* | 0.16 | 0.13 | 0.02 | 0.566 | NA |
| *Rauchvirus* | 0.16 | 0.55 | 0.13 | 0.38 | NA |
| *Rimavirus* | 0.16 | 0.10 | 0.03 | 0.566 | NA |
| *Pepyhexavirus* | 0.14 | 0.11 | 0.02 | 0.825 | NA |
| *Okubovirus* | 0.14 | 0.05 | 0.03 | 0.233 | NA |
| *Klosneuvirus* | 0.14 | 0.16 | 0.03 | 0.757 | NA |
| *Thornevirus* | 0.13 | 0.06 | 0.02 | 0.122 | NA |
| *Ackermannviridae_norank* | 0.12 | 0.13 | 0.02 | 0.825 | NA |
| *Skunavirus* | 0.12 | 0.14 | 0.01 | 0.508 | NA |
| *Pecentumvirus* | 0.12 | 0.07 | 0.03 | 0.825 | NA |
| *Bingvirus* | 0.12 | 0.08 | 0.02 | 0.566 | NA |
| *Caeruleovirus* | 0.11 | 0.06 | 0.01 | 0.122 | NA |
| *Cbastvirus* | 0.10 | 0.06 | 0.04 | 0.354 | NA |
| *Kayvirus* | 0.10 | 0.13 | 0.01 | 0.354 | NA |
| *Saphexavirus* | 0.09 | 0.08 | 0.01 | 0.508 | NA |
| *Aphroditevirus* | 0.09 | 0.08 | 0.02 | 0.452 | NA |
| *Tidunavirus* | 0.09 | 0.13 | 0.03 | 0.691 | NA |
| *Schizotequatrovirus* | 0.09 | 0.14 | 0.02 | 0.508 | NA |
| *Iridoviridae_norank* | 0.08 | 0.08 | 0.01 | 0.757 | NA |
| *Mudcatvirus* | 0.08 | 0.06 | 0.01 | 0.965 | NA |
| *Siminovitchvirus* | 0.08 | 0.03 | 0.03 | 0.2 | NA |
| *Obolenskvirus* | 0.08 | 0.05 | 0.01 | 0.27 | NA |
| *Eneladusvirus* | 0.08 | 0.05 | 0.01 | 0.27 | NA |
| *Dhillonvirus* | 0.08 | 0.16 | 0.04 | 0.627 | NA |
| *Rigallicvirus* | 0.08 | 0.00 | 0.04 | 0.146 | NA |
| *Galaxyvirus* | 0.08 | 0.03 | 0.02 | 0.31 | NA |
| *Mosigvirus* | 0.07 | 0.06 | 0.02 | 0.757 | NA |
| *Moineauvirus* | 0.07 | 0.10 | 0.02 | 0.691 | NA |
| *Chivirus* | 0.07 | 0.30 | 0.07 | 0.339 | NA |
| *Efquatrovirus* | 0.07 | 0.07 | 0.02 | 0.965 | NA |
| *Catovirus* | 0.06 | 0.09 | 0.02 | 0.825 | NA |
| *Marseilleviridae_norank* | 0.05 | 0.07 | 0.01 | 0.402 | NA |
| *Pamexvirus* | 0.05 | 0.15 | 0.06 | 0.508 | NA |
| *Tupanvirus* | 0.05 | 0.04 | 0.01 | 0.354 | NA |
| *Svunavirus* | 0.05 | 0.02 | 0.01 | 0.402 | NA |
| *Prasinovirus* | 0.05 | 0.04 | 0.01 | 0.895 | NA |
| *Vequintavirus* | 0.05 | 0.04 | 0.01 | 0.825 | NA |
| *Inhavirus* | 0.04 | 0.02 | 0.01 | 0.566 | NA |
| *Dhakavirus* | 0.04 | 0.03 | 0.01 | 0.757 | NA |
| *Incheonvrus* | 0.04 | 0.04 | 0.01 | 0.566 | NA |
| *Nipunavirus* | 0.04 | 0.16 | 0.03 | 0.171 | NA |
| *Tijeunavirus* | 0.03 | 0.04 | 0.01 | 0.825 | NA |
| *Pakpunavirus* | 0.03 | 0.01 | 0.01 | 0.2 | NA |
| *Fletchervirus* | 0.03 | 0.06 | 0.01 | 0.508 | NA |
| *Jwalphavirus* | 0.03 | 0.05 | 0.01 | 0.659 | NA |
| *Ahduovirus* | 0.03 | 0.18 | 0.04 | 0.163 | NA |
| *Inoviridae_norank* | 0.03 | 0.02 | 0.01 | 0.529 | NA |
| *Marseillevirus* | 0.03 | 0.01 | 0.01 | 0.309 | NA |
| *Alcyoneusvirus* | 0.03 | 0.04 | 0.01 | 0.825 | NA |
| *Chakrabartyvirus* | 0.02 | 0.13 | 0.04 | 0.354 | NA |
| *Cervidpoxvirus* | 0.02 | 0.04 | 0.01 | 0.895 | NA |
| *Marthavirus* | 0.02 | 0.00 | 0.01 | 0.249 | NA |
| *Tsarbombavirus* | 0.02 | 0.02 | 0.01 | 0.54 | NA |
| *Bequatrovirus* | 0.02 | 0.00 | 0.01 | 0.16 | NA |
| *Teseptimavirus* | 0.02 | 0.10 | 0.02 | 0.024 | 2.03 |
| *Lightbulbvirus* | 0.02 | 0.04 | 0.01 | 0.2 | NA |
| *Baltimorevirus* | 0.02 | 0.01 | 0.01 | 0.894 | NA |
| *Gelderlandvirus* | 0.02 | 0.01 | 0.01 | 0.529 | NA |
| *Jerseyvirus* | 0.02 | 0.00 | 0.01 | 0.131 | NA |
| *Gamaleyavirus* | 0.01 | 0.06 | 0.01 | 0.399 | NA |
| *Mieseafarmvirus* | 0.01 | 0.03 | 0.01 | 0.965 | NA |
| *Asteriusvirus* | 0.01 | 0.01 | 0.01 | 0.363 | NA |
| *Lokivirus* | 0.01 | 0.04 | 0.01 | 0.37 | NA |
| *Firehammervirus* | 0.01 | 0.05 | 0.02 | 0.884 | NA |
| *Tulanevirus* | 0.01 | 0.04 | 0.01 | 0.177 | NA |
| *Mardecavirus* | 0.01 | 0.02 | 0.01 | 1 | NA |
| *Vidavervirus* | 0.00 | 0.02 | 0.01 | 0.696 | NA |
| *Winklervirus* | 0.00 | 0.05 | 0.02 | 0.713 | NA |
| *Yuavirus* | 0.00 | 0.02 | 0.01 | 0.191 | NA |
| *Decurrovirus* | 0.00 | 0.02 | 0.01 | 0.099 | NA |
| *Iapetusvirus* | 0.00 | 0.03 | 0.01 | 0.145 | NA |
| *Gammaretrovirus* | 0.00 | 0.29 | 0.10 | 0.468 | NA |
| *Phicbkvirus* | 0.00 | 0.02 | 0.01 | 0.146 | NA |
| *Trichovirus* | 0.00 | 0.02 | 0.01 | 0.317 | NA |

NA: Not Applicable.

Only Phyla and genera with LDA Score >2 are displayed.
