# Supplementary material for: Metagenomic and Metabolomic Insights Into the Mechanism Underlying the Disparity in Milk Yield of Holstein Cows
Source: Front Microbiol. 2022 May 20;13:844968. doi: 10.3389/fmicb.2022.844968 (PMC9163737; doi:10.3389/fmicb.2022.844968)
Supplement: Supplementary file 9 [file Table_9.DOCX]

**Table S9a: Differential metabolites in the Serum**

| **S/N** | **Classification** | **Compounds** | **RT (Min)** | **Molecular Mass** | **VIP** | ***P*-value** | **Fold Change (HP/LP)** |
| --- | --- | --- | --- | --- | --- | --- | --- |
| 1 | Alkaloid | Pipecolic acid | 1.86 | 129.08 | 1.15 | 0.041 | 1.179 |
| 2 | Amines | Tetradecylamine | 6.30 | 213.25 | 2.04 | <0.001 | 1.142 |
| 3 | Amines | Stearoylethanolamide | 11.38 | 327.31 | 1.72 | 0.002 | 1.310 |
| 4 | Amines | Palmitoylethanolamide | 10.00 | 299.28 | 1.65 | 0.003 | 1.220 |
| 5 | Amines | N,N-Dimethylaniline | 17.50 | 121.09 | 1.68 | 0.004 | 1.310 |
| 6 | Amines | Spermidine | 1.39 | 145.16 | 1.63 | 0.006 | 2.233 |
| 7 | Amines | Oleoylethanolamide | 10.24 | 325.30 | 1.34 | 0.037 | 1.499 |
| 8 | Amines | Tyramine | 1.54 | 137.08 | 1.37 | 0.038 | 1.318 |
| 9 | Amino acids and their derivatives | Methionine sulfoxide | 1.56 | 165.05 | 1.92 | <0.001 | 1.511 |
| 10 | Amino acids and their derivatives | N6-Acetyl-L-lysine | 1.67 | 188.12 | 1.81 | 0.001 | 1.277 |
| 11 | Amino acids and their derivatives | Histamine | 1.37 | 111.08 | 1.54 | 0.002 | 1.599 |
| 12 | Amino acids and their derivatives | 5-Aminopentanoic acid | 1.52 | 117.08 | 1.60 | 0.006 | 0.757 |
| 13 | Amino acids and their derivatives | L-Methionine | 1.87 | 149.05 | 1.53 | 0.008 | 1.377 |
| 14 | Amino acids and their derivatives | Creatinine | 1.52 | 113.06 | 1.43 | 0.017 | 1.177 |
| 15 | Amino acids and their derivatives | N-Acetylornithine | 1.53 | 174.10 | 1.29 | 0.021 | 1.424 |
| 16 | Amino acids and their derivatives | L-Homotyrosine | 3.54 | 195.09 | 1.35 | 0.021 | 1.399 |
| 17 | Amino acids and their derivatives | L-Norleucine | 1.56 | 131.09 | 1.34 | 0.027 | 1.286 |
| 18 | Amino acids and their derivatives | 5-Methoxytryptophan | 4.37 | 234.10 | 1.35 | 0.028 | 0.629 |
| 19 | Amino acids and their derivatives | Norvaline | 1.25 | 117.08 | 1.31 | 0.029 | 1.374 |
| 20 | Amino acids and their derivatives | DL-ß-Leucine | 1.65 | 131.09 | 1.15 | 0.049 | 1.344 |
| 21 | Arachidonic acid | 8-iso-15-keto-PGF2a | 4.66 | 352.23 | 1.29 | 0.037 | 1.100 |
| 22 | Bases | Uracil | 1.55 | 112.03 | 1.73 | 0.002 | 1.403 |
| 23 | Bases | Cytosine | 1.53 | 111.04 | 1.60 | 0.007 | 1.322 |
| 24 | Benzoic acids and their derivatives | p-Aminobenzoic acid | 1.08 | 137.05 | 1.46 | 0.016 | 1.345 |
| 25 | Bilirubin | Bilirubin | 11.67 | 584.26 | 1.72 | 0.002 | 2.052 |
| 26 | Carnitines | Stearoylcarnitine | 9.00 | 427.37 | 1.67 | 0.002 | 1.748 |
| 27 | Carnitines | Oleoylcarnitine | 8.35 | 425.35 | 1.60 | 0.004 | 1.955 |
| 28 | Carnitines | L-Palmitoylcarnitine | 8.17 | 399.33 | 1.45 | 0.012 | 1.640 |
| 29 | Cinnamic Acids | Cinnamic acid | 2.68 | 148.05 | 1.31 | 0.046 | 1.159 |
| 30 | Fatty acids | Docosapentaenoic acid (22n-6) | 10.72 | 330.26 | 2.01 | <0.001 | 1.810 |
| 31 | Fatty acids | Dihomo-gamma-linolenic acid | 11.13 | 306.26 | 1.87 | <0.001 | 2.075 |
| 32 | Fatty acids | a-Linolenic acid | 10.00 | 278.22 | 1.84 | 0.001 | 1.517 |
| 33 | Fatty acids | Arachidonic acid | 10.56 | 304.24 | 1.76 | 0.002 | 1.742 |
| 34 | Fatty acids | Linoleic acid | 9.33 | 280.24 | 1.60 | 0.004 | 1.409 |
| 35 | Fatty acids | Palmitic acid | 11.08 | 256.24 | 1.54 | 0.005 | 2.130 |
| 36 | Fatty acids | Stearic acid | 12.38 | 284.27 | 1.63 | 0.005 | 1.862 |
| 37 | Fatty acids | Palmitoleic acid | 7.79 | 254.22 | 1.27 | 0.014 | 1.403 |
| 38 | Fatty acids | Isobutyric acid | 3.78 | 88.05 | 1.33 | 0.027 | 1.348 |
| 39 | Fatty acids | (9E,11E)-Octadecadienoic acid | 10.33 | 280.24 | 1.11 | 0.038 | 1.256 |
| 40 | Imidazoles | Allantoin | 1.52 | 158.04 | 1.88 | 0.001 | 1.241 |
| 41 | Indoles | Indoleacetic acid | 4.72 | 175.06 | 1.62 | 0.008 | 1.507 |
| 42 | Indoles | 5-Hydroxyindoleacetic acid | 3.86 | 191.06 | 1.26 | 0.040 | 1.276 |
| 43 | Lipids | LysoPE(18:0/0:0) | 9.47 | 481.32 | 1.92 | <0.001 | 1.296 |
| 44 | Lipids | LysoPC(16:0) | 9.38 | 495.33 | 1.82 | 0.001 | 1.260 |
| 45 | Lipids | LysoPC(15:0/0:0) | 8.50 | 481.32 | 1.54 | 0.013 | 1.331 |
| 46 | Lipids | Glycerophosphocholine | 1.52 | 257.10 | 1.65 | 0.031 | 1.543 |
| 47 | Nucleosides | 2'-Deoxyuridine | 1.46 | 228.07 | 1.62 | 0.005 | 1.519 |
| 48 | Nucleosides | Deoxyinosine | 1.50 | 252.09 | 1.46 | 0.008 | 1.283 |
| 49 | Nucleosides | Uridine | 1.43 | 244.07 | 1.56 | 0.008 | 1.350 |
| 50 | Nucleosides | Cytidine | 1.51 | 243.09 | 1.68 | 0.009 | 1.474 |
| 51 | Nucleosides | Inosine | 1.37 | 268.08 | 1.39 | 0.018 | 1.352 |
| 52 | Nucleosides | Cyclic AMP | 1.55 | 329.05 | 1.56 | 0.008 | 1.152 |
| 53 | Organic acids | 2-Ketobutyric acid | 1.45 | 102.03 | 1.56 | 0.007 | 1.392 |
| 54 | Organic acids | 12,13-DHOME | 6.90 | 314.25 | 1.15 | 0.048 | 1.326 |
| 55 | Organic acids | Tetradecanedioic acid | 5.81 | 258.18 | 1.61 | 0.002 | 1.646 |
| 56 | Organic acids | Dodecanedioic acid | 5.05 | 230.15 | 1.69 | 0.003 | 1.312 |
| 57 | Organic acids | Hexadecanedioic acid | 7.00 | 286.21 | 1.68 | 0.003 | 1.362 |
| 58 | Organic acids | 3-Hydroxysebacic acid | 3.94 | 218.12 | 1.37 | 0.011 | 1.299 |
| 59 | Organic acids | Ureidopropionic acid | 1.86 | 132.05 | 1.48 | 0.013 | 1.438 |
| 60 | Organic acids | Pyruvate | 1.43 | 88.02 | 1.44 | 0.020 | 1.281 |
| 61 | Organic acids | trans-Aconitic acid | 1.43 | 174.02 | 1.40 | 0.022 | 1.224 |
| 62 | Organic acids | Lactic acid | 1.13 | 90.03 | 1.34 | 0.022 | 1.493 |
| 63 | Organic acids | 2-Hydroxystearic acid | 8.48 | 300.27 | 1.35 | 0.027 | 1.285 |
| 64 | Organic acids | 3-Methyladipic acid | 3.73 | 160.07 | 1.31 | 0.034 | 1.221 |
| 65 | Organic acids | 2-Hydroxymyristic acid | 8.41 | 244.20 | 1.23 | 0.037 | 1.179 |
| 66 | Others | Indoxyl sulfate | 3.74 | 213.01 | 1.77 | 0.001 | 1.482 |
| 67 | Others | N-Acetylhistamine | 1.52 | 153.09 | 1.51 | 0.013 | 1.336 |
| 68 | Peptides | Alanylleucine | 1.53 | 202.13 | 1.50 | 0.011 | 1.298 |
| 69 | Phenols | Dopamine | 1.54 | 153.08 | 1.33 | 0.036 | 1.238 |
| 70 | Phenols | Acetaminophen | 1.55 | 151.06 | 1.43 | 0.044 | 1.450 |
| 71 | Steroids | Aldosterone | 5.76 | 360.19 | 1.44 | 0.007 | 1.533 |
| 72 | Steroid | 7-Ketocholesterol | 12.43 | 400.33 | 1.45 | 0.018 | 1.950 |
| 73 | Steroids | beta-Sitosterol | 8.63 | 414.39 | 1.03 | 0.043 | 1.422 |
| 74 | Sugars and their derivatives | D-Xylose | 1.87 | 150.05 | 1.43 | 0.012 | 1.311 |
| 75 | Sugars and their derivatives | Erythrose | 1.87 | 120.04 | 1.33 | 0.031 | 1.136 |
| 76 | Vitamins | 4-Pyridoxic acid | 1.58 | 183.05 | 1.75 | 0.002 | 1.162 |
| 77 | Vitamins | Pyridoxine | 1.53 | 169.07 | 1.73 | 0.004 | 1.388 |
| 78 | Vitamins | Nicotinic acid | 1.86 | 123.03 | 1.40 | 0.037 | 2.471 |

**Table S9b: Differential metabolites in the Rumen fluid**

| **S/N** | **Classification** | **Compounds** | **RT (Min)** | **Molecular Mass** | **VIP** | ***P*-value** | **Fold Change (HP/LP)** |
| --- | --- | --- | --- | --- | --- | --- | --- |
| 1 | Amines | Phenylethylamine | 3.90 | 121.09 | 1.68 | 0.035 | 1.57 |
| 2 | Amines | N,N-Dimethylaniline | 17.50 | 121.09 | 2.55 | 0.003 | 1.27 |
| 3 | Bilirubin | Bilirubin | 12.12 | 584.26 | 2.14 | 0.020 | 1.08 |
| 4 | Carnitines | Oleoylcarnitine | 8.35 | 425.35 | 2.33 | 0.008 | 1.09 |
| 5 | Carnitines | Stearoylcarnitine | 9.00 | 427.37 | 2.02 | 0.012 | 0.61 |
| 6 | Carnitines | L-Hexanoylcarnitine | 4.25 | 259.18 | 1.96 | 0.026 | 0.83 |
| 7 | Fatty acid | Lauric acid | 6.07 | 200.18 | 1.89 | 0.046 | 0.93 |
| 8 | Lipids | LysoPC(15:0/0:0) | 8.50 | 481.32 | 1.91 | 0.030 | 1.11 |
| 9 | Lipids | 13-OxoODE | 7.80 | 294.22 | 1.83 | 0.036 | 2.03 |
| 10 | Lipids | 9(S)-HPODE | 7.81 | 312.23 | 1.83 | 0.040 | 2.08 |
| 11 | Organic acids | 3-Hydroxymethylglutaric acid | 1.51 | 162.05 | 2.04 | 0.021 | 1.36 |
| 12 | Organic acids | 2-Hydroxymyristic acid | 8.41 | 244.20 | 1.98 | 0.022 | 0.70 |
| 13 | Others | Kynurenic acid | 4.06 | 189.04 | 2.00 | 0.020 | 1.79 |
| 14 | Phenols | Pyrogallol | 1.54 | 126.03 | 3.05 | <0.001 | 1.11 |
| 15 | Phenols | Dopamine | 1.54 | 153.08 | 2.38 | 0.004 | 0.82 |
| 16 | Phenols | 3-Methoxytyramine | 4.14 | 167.09 | 2.29 | 0.010 | 0.71 |
| 17 | Phenols | Acetaminophen | 1.55 | 151.06 | 1.90 | 0.046 | 0.82 |
| 18 | Pyridines | Picolinic acid | 1.41 | 123.03 | 1.78 | 0.029 | 0.67 |
| 19 | Steroids | Chenodeoxycholic acid glycine conjugate | 6.25 | 449.31 | 2.77 | 0.001 | 1.09 |
| 20 | Steroid | Aldosterone | 5.76 | 360.19 | 2.07 | 0.032 | 0.75 |

**Table S9c: Differential metabolites in the milk**

| **S/N** | **Classification** | **Compounds** | **RT (Min)** | **Molecular Mass** | **VIP** | ***P*-value** | **Fold Change (HP/LP)** |
| --- | --- | --- | --- | --- | --- | --- | --- |
| 1 | Alkyl benzoketones | Kynurenine | 2.53 | 208.08 | 1.96 | 0.031 | 0.47 |
| 2 | Amine | Tetradecylamine | 6.30 | 213.25 | 2.63 | 0.001 | 1.28 |
| 3 | Amino acids and their derivatives | L-Valine | 1.79 | 117.08 | 2.00 | 0.017 | 0.69 |
| 4 | Amino acids and their derivatives | L-Homotyrosine | 3.54 | 195.09 | 1.97 | 0.019 | 0.81 |
| 5 | Amino acids and their derivatives | L-Lysine | 1.38 | 146.11 | 1.99 | 0.020 | 1.51 |
| 6 | Amino acids and their derivatives | 5-Aminopentanoic acid | 1.52 | 117.08 | 2.03 | 0.023 | 0.60 |
| 7 | Amino acids and their derivatives | Citrulline | 1.50 | 175.10 | 1.89 | 0.027 | 1.35 |
| 8 | Amino acids and their derivatives | gamma-Aminobutyric acid | 1.86 | 103.06 | 2.03 | 0.027 | 1.58 |
| 9 | Amino acids and their derivatives | N-Acetylornithine | 1.53 | 174.10 | 2.09 | 0.048 | 1.49 |
| 10 | Benzoic acids and their derivatives | Gentisic acid | 3.69 | 154.03 | 2.00 | 0.021 | 0.91 |
| 11 | Carnitine | Oleoylcarnitine | 8.35 | 425.35 | 1.75 | 0.011 | 0.42 |
| 12 | Fatty acids | Arachidonic acid | 10.56 | 304.24 | 2.36 | 0.006 | 0.72 |
| 13 | Fatty acids | Lauric acid | 6.07 | 200.18 | 2.19 | 0.007 | 0.86 |
| 14 | Fatty acids | Docosapentaenoic acid (22n-6) | 10.72 | 330.26 | 2.26 | 0.014 | 0.63 |
| 15 | Imidazoles | Allantoin | 1.09 | 158.04 | 1.95 | 0.039 | 1.40 |
| 16 | Lipids | LysoPE(P-16:0/0:0) | 8.47 | 437.29 | 2.37 | 0.003 | 0.77 |
| 17 | Lipid | LysoPE(15:0/0:0) | 6.33 | 439.27 | 2.21 | 0.009 | 0.82 |
| 18 | Nucleosides | Adenosine | 1.54 | 267.10 | 2.27 | 0.002 | 1.83 |
| 19 | Nucleoside | 7-Methylguanosine | 2.07 | 297.11 | 2.33 | 0.010 | 1.38 |
| 20 | Organic acids | Ureidopropionic acid | 1.86 | 132.05 | 2.02 | 0.019 | 1.27 |
| 21 | Others | Urocanic acid | 1.41 | 138.04 | 1.80 | 0.019 | 1.40 |
| 22 | Phenols | Dopamine | 1.54 | 153.08 | 2.00 | 0.020 | 0.83 |
| 23 | Steroids | beta-Sitosterol | 8.63 | 414.39 | 2.32 | 0.007 | 1.68 |
| 24 | Vitamin | D-Biotin | 4.19 | 244.09 | 2.44 | 0.001 | 0.66 |
