# Supplementary material for: Metagenomic and Metabolomic Insights Into the Mechanism Underlying the Disparity in Milk Yield of Holstein Cows
Source: Front Microbiol. 2022 May 20;13:844968. doi: 10.3389/fmicb.2022.844968 (PMC9163737; doi:10.3389/fmicb.2022.844968)
Supplement: Supplementary file 11 [file Table_11.DOCX]

Table S11a. KEGG Pathway enrichment by the Serum metabolites

| S/N | Pathway | Match status | Metabolites | Pathway impact | *P*-value | Group |
| --- | --- | --- | --- | --- | --- | --- |
| 1. | Pyrimidine metabolism | 5/38 | Uracil (C00106), Cytidine (C00475), Ureidopropionic acid (C02642), 2'-Deoxyuridine (C00526), Uridine (C00299) | 0.20 | 0.014 | HP |
| 2. | beta-Alanine metabolism | 3/21 | Spermidine (C00315), Ureidopropionic acid (C02642), Uracil (C00106) | 0.10 | 0.046 | HP |

Table S11b. KEGG Pathway enrichment by the Milk metabolites

| S/N | Pathway | Match status | Metabolites | Pathway impact | *P*-value | Group |
| --- | --- | --- | --- | --- | --- | --- |
| 1. | Arginine biosynthesis | 2/14 | Citrulline (C00327), N-Acetylornithine (C00437) | 0.23 | 0.003 | HP |
| 2. | Tyrosine metabolism | 2/42 | Dopamine (C03758), Gentisic acid (C00628) | 0.13 | 0.019 | LP |

Table S11c. KEGG Pathway enrichment by the Rumen fluid metabolites

| S/N | Pathway | Match status | Metabolites | Pathway impact | *P-*value | Group |
| --- | --- | --- | --- | --- | --- | --- |
| 1. | Tyrosine metabolism | 2/42 | Dopamine (C03758), 3-Methoxytyramine (C05587) | 0.15 | 0.012 | LP |
