# Supplementary figures and images for: Metagenomic and Metabolomic Insights Into the Mechanism Underlying the Disparity in Milk Yield of Holstein Cows
Source: Front Microbiol. 2022 May 20;13:844968. doi: 10.3389/fmicb.2022.844968 (PMC9163737; doi:10.3389/fmicb.2022.844968)

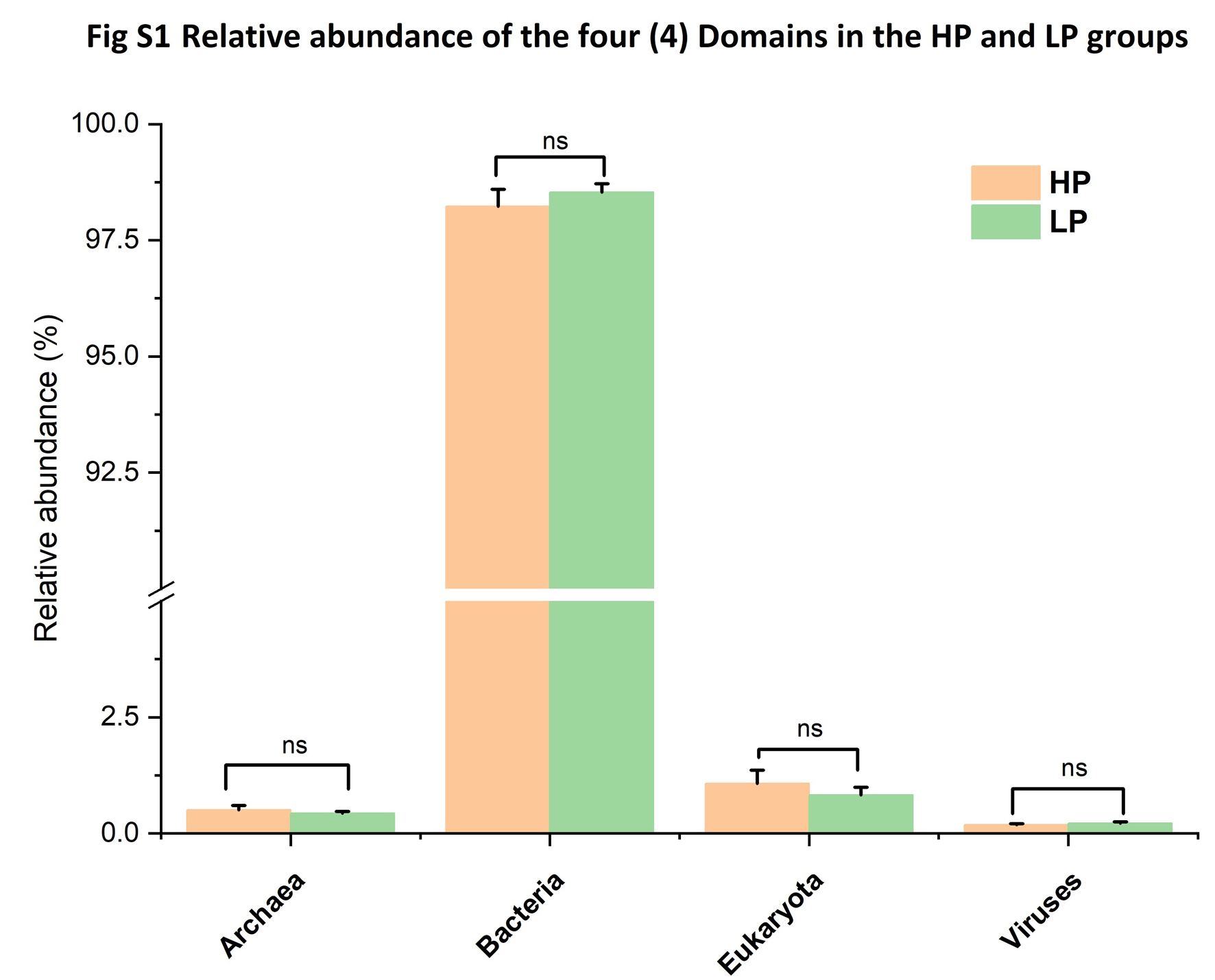

Supplement: Supplementary file 14 [file Image_1.JPEG]

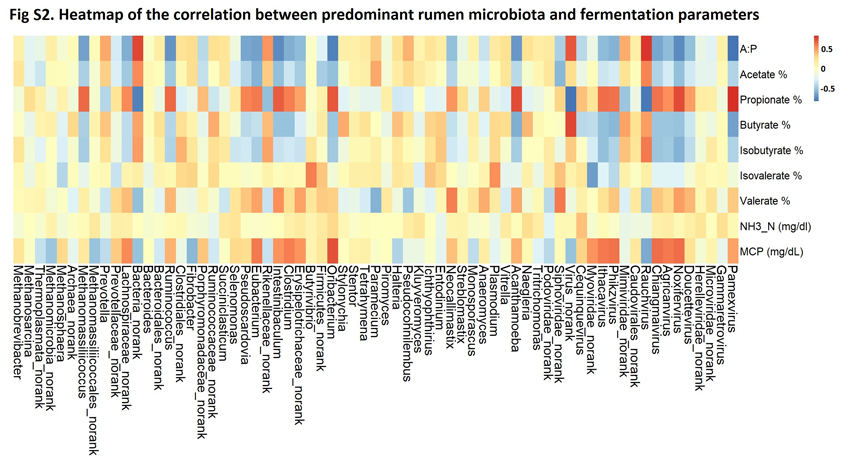

Supplement: Supplementary file 15 [file Image_2.JPEG]

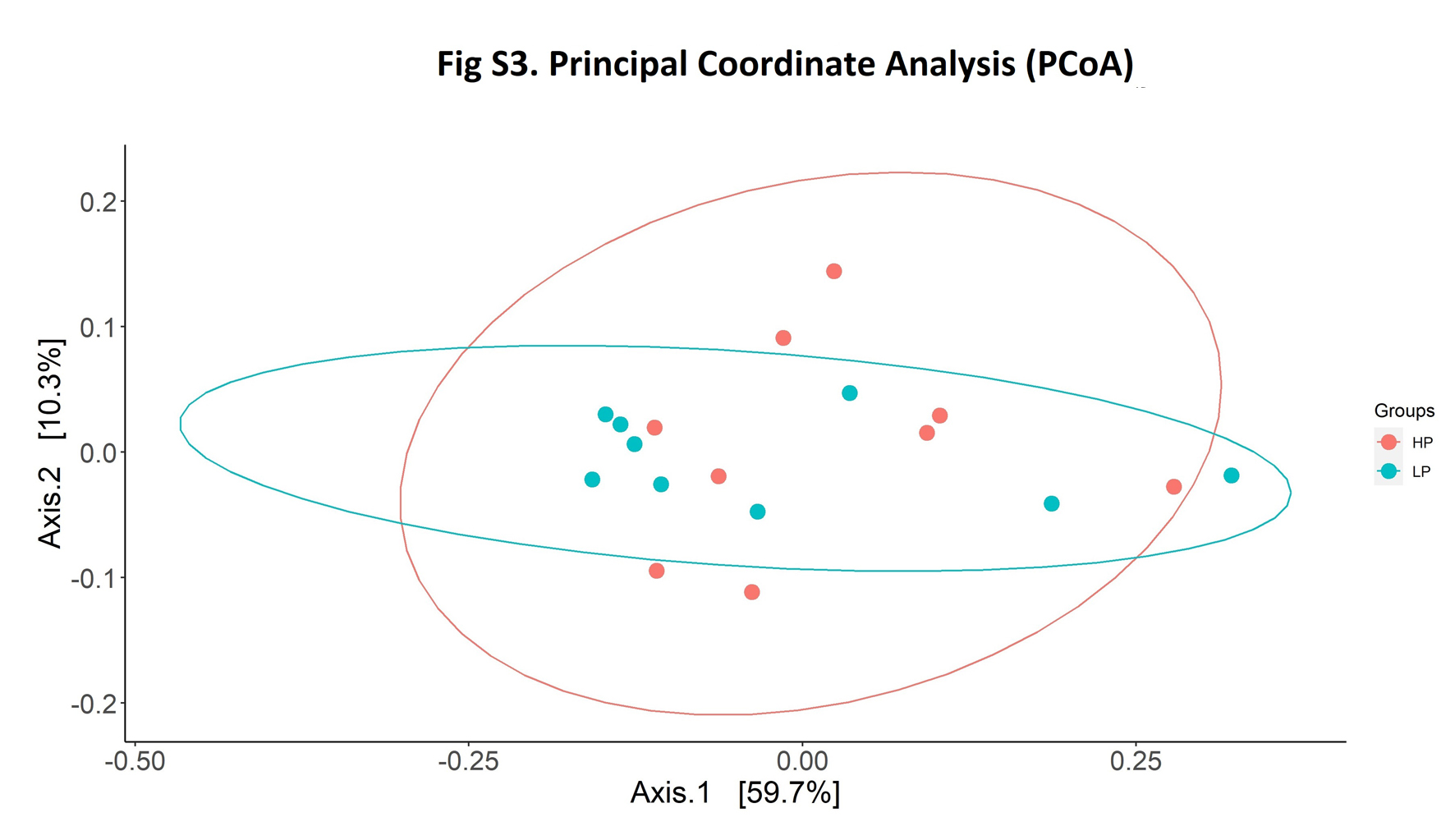

Supplement: Supplementary file 16 [file Image_3.JPEG]

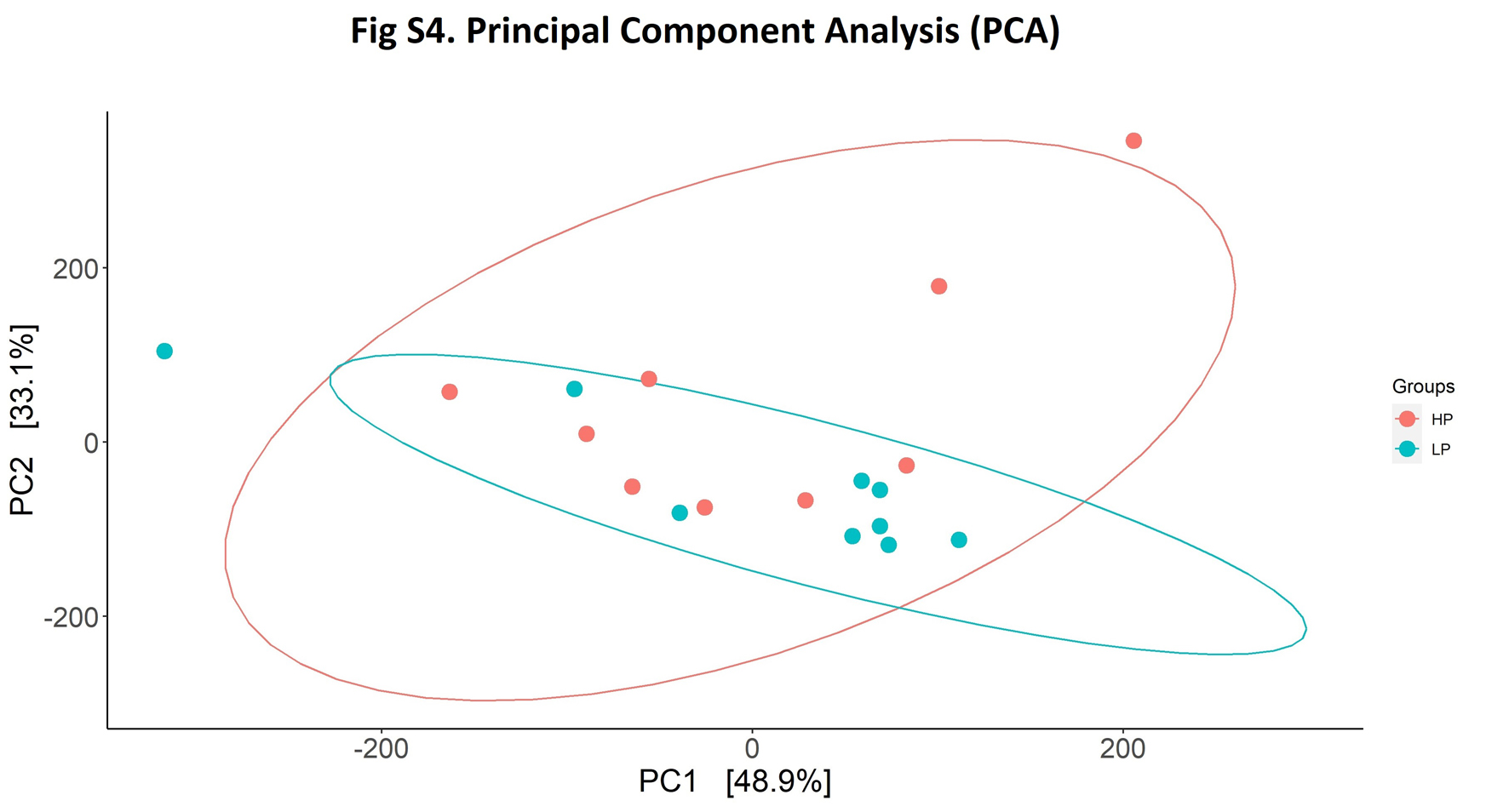

Supplement: Supplementary file 17 [file Image_4.JPEG]

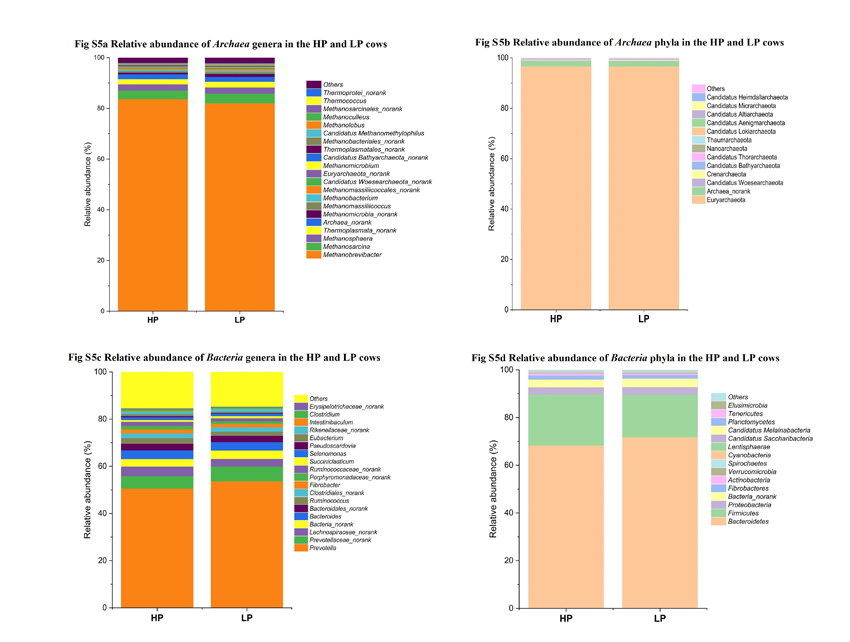

Supplement: Supplementary file 18 [file Image_5.JPEG]

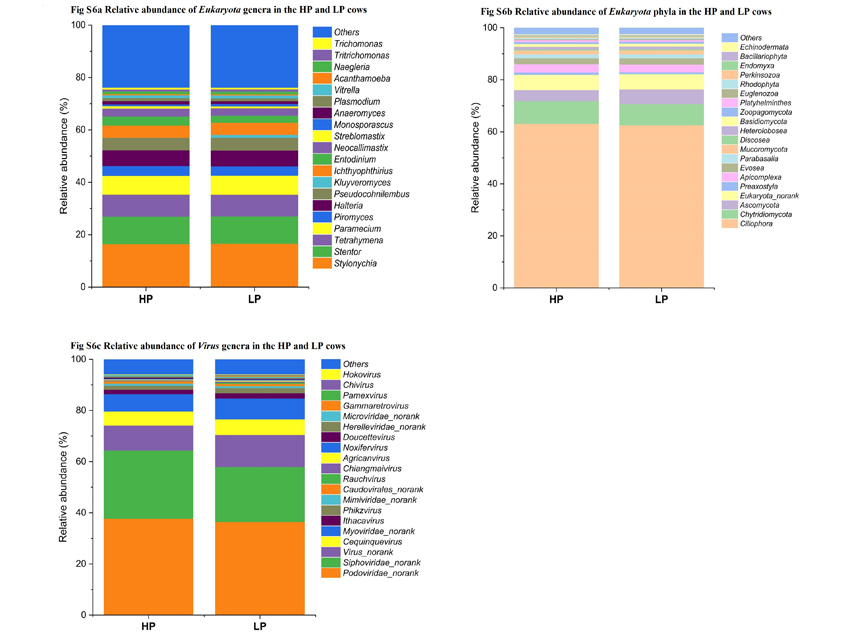

Supplement: Supplementary file 19 [file Image_6.JPEG]

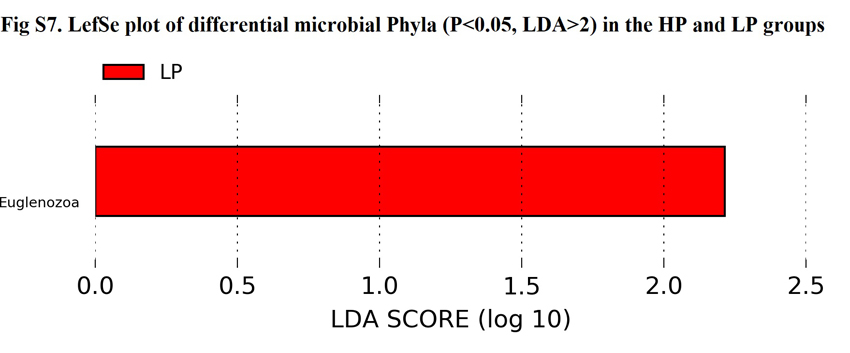

Supplement: Supplementary file 20 [file Image_7.JPEG]
